# Bifunctional zeolites-silver catalyst enabled tandem oxidation of formaldehyde at low temperatures

**DOI:** 10.1038/s41467-022-29936-8

**Published:** 2022-04-22

**Authors:** Na Li, Bin Huang, Xue Dong, Jinsong Luo, Yi Wang, Hui Wang, Dengyun Miao, Yang Pan, Feng Jiao, Jianping Xiao, Zhenping Qu

**Affiliations:** 1 Key Laboratory of Industrial Ecology and Environmental Engineering (Ministry of Education, China), School of Environmental Science and Technology, Dalian University of Technology, 2 Linggong Road, Dalian, 116024 China; 2 Dalian Institute of Chemical Physics, Chinese Academy of Sciences, 457 Zhongshan Road, Dalian, 116023 China; 3 National Synchrotron Radiation Laboratory, University of Science and Technology of China, 96 Jinzhai Road, Hefei, 230026 China

**Keywords:** Heterogeneous catalysis, Pollution remediation, Catalytic mechanisms

## Abstract

Bifunctional catalysts with tandem processes have achieved great success in a wide range of important catalytic processes, however, this concept has hardly been applied in the elimination of volatile organic compounds. Herein, we designed a tandem bifunctional Zeolites-Silver catalyst that enormously boosted formaldehyde oxidation at low temperatures, and formaldehyde conversion increased by 50 times (100% *versus* 2%) at 70 °C compared to that of monofunctional supported silver catalyst. This is enabled by designing a bifunctional catalyst composed of acidic ZSM-5 zeolite and silver component, which provides two types of active sites with complementary functions. Detached acidic ZSM-5 activates formaldehyde to generate gaseous intermediates of methyl formate, which is more easily oxidized by subsequent silver component. We anticipate that the findings here will open up a new avenue for the development of formaldehyde oxidation technologies, and also provide guidance for designing efficient catalysts in a series of oxidation reactions.

## Abstract (web summary)

Formaldehyde oxidation suffers from insufficient activity at low temperatures due to the lack of efficient silver catalyst. Herein, the authors design a tandem bifunctional zeolites-silver catalyst that achieves a 50 times improvement in activity (100% *versus* 2%) at 70 °C as compared to monofunctional supported silver catalyst.

## Introduction

Formaldehyde (HCHO), one of the major indoor and industrial pollutants, is extremely harmful to the environment and humans’ health. Therefore, it is important to develop effective catalysts to oxidize HCHO into nontoxic CO_2_ and H_2_O^1,2^. So far, most studies about HCHO oxidation have focused on supported noble metal catalysts and metal oxide catalysts^3–5^. Although significant progress has been made by adjusting their structures, compositions, and synthesized conditions^6–9^, the catalytic activity still needs further improvement. Therefore, new concepts for designing more effective catalysts are desirable. Multifunctional catalysts have distinct types of active sites. The synergy between these active sites has made a crucial contribution to improving the catalytic activity and unexpected product selectivity in a series of important catalytic processes^10–13^. For instance, Yang and coworkers^14^ designed a nanocrystal bilayer structure containing two distinct metal–metal oxide interfaces, CeO_2_–Pt and Pt–SiO_2_, which enabled a tandem reaction, including methanol decomposition to CO and H_2_ process and ethylene hydroformylation to propanal process. Ultimately, the selective synthesis of propanal was achieved directly from methanol and ethylene over the bifunctional nanocrystal catalyst. Bao and coworkers^10^ proposed a bifunctional oxide–zeolite (OXZEO) concept, which separated CO activation and C–C coupling onto two different active sites, thereby achieving surprisingly high-light olefin selectivity directly from syngas. Similar bifunctional catalyst-design strategy has also been proved to be effective in CO_2_ hydrogenation to value-added chemicals, such as liquid fuels, with high selectivities^11,13,15–18^.

In bifunctional catalyst, the activity and selectivity can be optimized by adjusting the match and intimacy between their active sites^10,19–22^. de Jong and coworkers^19^ investigated that the bifunctional catalyst consisted of zeolite Y and alumina binder, with platinum metal controllably deposited on either zeolite or binder to adjust the proximity between metal and zeolite acid sites. Their study showed that the selectivity of diesel from cracking large hydrocarbon molecules could be optimized at a nanoscale rather than closest intimacy. Bao and coworkers^10,22^ also reported a proximity-dependent syngas-to-light olefin process over OXZEO catalysts. They found that the activity increased with the proximity of ZnCrO_x_ and SAPO-34 due to the reduced mass transport limitation and it leveled off at the nanoscale proximity. However, the light olefin selectivity exhibits an optimum with the proximity, and nanoscale proximity is detrimental because of the segregation and migration of zinc species under reaction conditions^22^.

Inspired by the strategy of constructing and separating multiple active centers, we design herein a bifunctional catalyst composed of acidic ZSM-5 zeolite and supported silver (Ag) nanoparticles, which achieves the catalytic oxidation of HCHO at low temperatures via a tandem mode. Considering the millimeter-sized intimacy between zeolite and Ag components, gaseous intermediates are proved to exist and connect the entire process. Due to the easier oxidation of intermediates than HCHO over the following Ag component, the activity is significantly enhanced compared to that over traditional supported Ag nanoparticles. In addition, a novel tandem reaction mechanism via methyl formate gas-phase intermediate is proposed for this HCHO oxidation tandem process instead of classical Langmuir–Hinshelwood (L–H) mechanism over previously supported monofunctional metal catalysts^23–26^. In the L–H mechanism, the reaction began with adsorbed HCHO and O_2_ molecules, whose interaction produced typical surface-adsorbed species such as dioxymethylene (DOM), formate, and CO, which were eventually converted to CO_2_ through a variety of pathways with most evolutions taking place on catalyst surfaces^23–26^. This study opens up a new avenue for the development of volatile organic compound (VOCs) elimination technology and provides a broad prospect for the industrial application of silver-based catalysts.

## Results

### Catalytic performance of ZSM-5−Ag catalyst

**Fig. 1:**
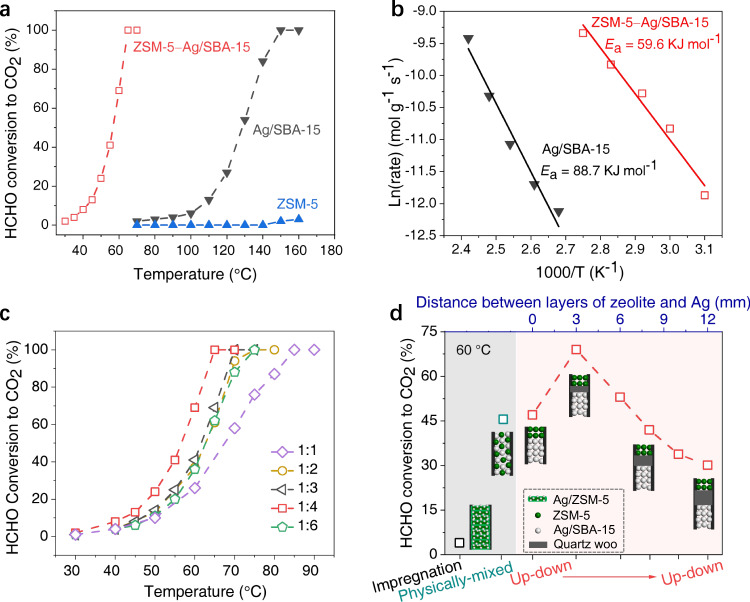
Bifunctionality of ZSM-5−Ag/SBA-15 catalyst in HCHO conversion. **a** HCHO conversion to CO_2_. **b** Reaction activation energy, *E*_a_. Individual ZSM-5 and Ag/SBA-15 components are listed for comparison. Effect of (**c**) mass ratio of ZSM-5 zeolite to Ag/SBA-15 component and **d** intimacy of the two components in composites. Reaction conditions: 60 °C, 100 ppm HCHO, 20% O_2_–Ar, and gas hourly space velocity (GHSV) with respect to ZSM-5**−**Ag/SBA-15 bifunctional catalysts is 36000 mL g^−1^ h^−1^.

Mesoporous SBA-15-supported Ag (named as Ag/SBA-15) was synthesized using a traditional impregnation method with the Ag loading of 10 wt%. X-ray diffraction (XRD) pattern reveals a typical Ag phase with the JCPDS number of 04-0783 (Supplementary Fig. 1). The average crystal size of Ag estimated using the Scherrer equation is 14 nm. Transmission electron microscopy (TEM) images show the well-dispersed Ag nanoparticles on SBA-15 carrier (Supplementary Fig. 2). Figure 1a demonstrates that Ag/SBA-15 alone gives inferior activity below 100 °C and the complete HCHO oxidation temperature reaches 150 °C (gray triangle) under conditions of 100 ppm HCHO, 20% O_2_–Ar, and gas hourly space velocity (GHSV) = 36000 mL g^−1^ h^−^^1^. Interestingly, upon addition of acid ZSM-5 zeolite (SiO_2_/Al_2_O_3_ = 46, JCPDS no. 37-0359 in Supplementary Fig. 3) in front of Ag/SBA-15 layer at a distance of 3 mm separated by inert quartz wool, the low-temperature activity is significantly enhanced, and the complete oxidation temperature is dramatically reduced to 65 °C (red hollow square), decreased by 85 °C. HCHO conversion of the ZSM-5−Ag/SBA-15 catalyst is 50 times higher than that over Ag/SBA-15 alone at 70 °C (100% vs. 2%). Furthermore, no obvious deactivation is observed within 35 hours on stream for this dual-bed ZSM-5−Ag catalyst (Supplementary Fig. 4). Notably, acidic ZSM-5 alone has almost no activity for HCHO oxidation to CO_2_ below 160 °C under the same reaction conditions (blue triangle), and meanwhile, putting acidic ZSM-5 zeolite under Ag/SBA-15 layer also gives poor activity, similar to that of Ag/SBA-15 alone (Supplementary Fig. 5). Therefore, these results indicate that acidic ZSM-5 zeolite here plays a pivotal role in the activation of HCHO to produce active gaseous intermediates, which can be transferred to subsequent Ag component and further be oxidized to CO_2_. This tandem process is similar to those over other bifunctional catalysts such as ZnCrO_x_/SAPO-34, In_2_O_3_/HZSM-5, and Co/Y_meso_ reported in CO/CO_2_ hydrogenation^10–12,27^. Macroscopic kinetics studies exhibit that the apparent activation energy (*E*_a_) of HCHO conversion to CO_2_ over bifunctional ZSM-5−Ag/SBA-15 catalyst is obviously lower than that over Ag/SBA-15 alone (Fig. 1b, 59.6 vs, 88.7 KJ mol^−^^1^), further demonstrating the superiority of the bifunctional catalyst.

The bifunctionality was further investigated. Figure 1c shows that HCHO conversion increases with Ag/SBA-15 content below 80 wt% in composites at 30−85 °C. For instance, the complete conversion temperature is only 85 °C at 50 wt% Ag/SBA-15 content (ratio of 1:1), while it decreases to 75 °C at 67 wt% Ag/SBA-15 (1:3), and further reduces to 65 °C at 80 wt% Ag/SBA-15 (1:4). Nevertheless, further enhancing Ag/SBA-15 content to 86 wt% (1:6), HCHO conversion decreases obviously with the complete conversion temperature rising to 75 °C again. This is mainly due to the mismatch between the two components caused by the insufficient ZSM-5 and excessive Ag component, which leads to insufficient active sites for HCHO activation to more active intermediates over the upper ZSM-5 component, thereby reducing the overall reaction performance of bifunctional catalysts. This phenomenon is general and has been reported in other bifunctional catalysts for syngas conversion and CO_2_ hydrogenation^10–13^. The results here demonstrate the important role of synergy effect of the two functions, and 80 wt% Ag/SBA-15 is adequate for the study of the individual role in composites.

Figure 1d exhibits that HCHO conversion varies distinctly as the intimacy of the two components at 60 °C. Intriguingly, the closest intimacy with Ag impregnated on ZSM-5 (Ag/ZSM-5) only gives 5% HCHO conversion (black hollow square), while it reaches 45% upon decreasing the intimacy by physically mixing the two components (cyan hollow square). Further reducing the intimacy by separating the two components with inert quartz wool (up–down mode with zeolite on the upper layer) shows an obvious dependence on activity (red hollow square). For instance, the direct contact of ZSM-5 layer with Ag/SBA-15 layer (marked 0 mm distance) gives 47% HCHO conversion, whereas it significantly enhances to 70% at a distance of 3 mm. However, further enlarging the distance to 6 and 12 mm, HCHO conversion decreases to 55% and 30% separately. To well elucidate the reasons for the decreased activity over long distances, we have further conducted mass transfer-dependent catalytic activity at a selected interval distance of 6 mm between ZSM-5 and Ag/SBA-15. By changing the weight of quartz wool, the packing density of quartz wool and thereby the mass transfer ability of intermediates can be modulated. As displayed in Supplementary Fig. 6, the quartz wool weight does affect the overall catalytic activity of HCHO conversion. Compared with loading 6 mg of quartz wool at the same height of 6 mm, loading 10 mg of quartz wool can reduce the HCHO conversion from 51% to 48%. Further increasing the weight of quartz wool (i.e., the density of quartz wool), HCHO conversion is further reduced, 38% at 12 mg and 35% at 14 mg. These results clearly demonstrated the important role of mass transfer of intermediates on turning the overall performance of this tandem process. Such mass transfer effect in tandem process was also observed and proposed as the “intimacy criterion”, which was first proposed by Weisz on investigating isomerization and hydrocracking^28^. A feature of “the closer, the better” was well demonstrated and widely accepted in the field of tandem reaction considering the diffusion limitation of reaction intermediates over multifunctional catalysts^10,11^. Here in our case, 3 mm distance should give better mass transfer of intermediates than 6 mm distance and therefore lead to much higher HCHO conversion.

### Reaction intermediates and active sites of ZSM-5 zeolite

**Fig. 2:**
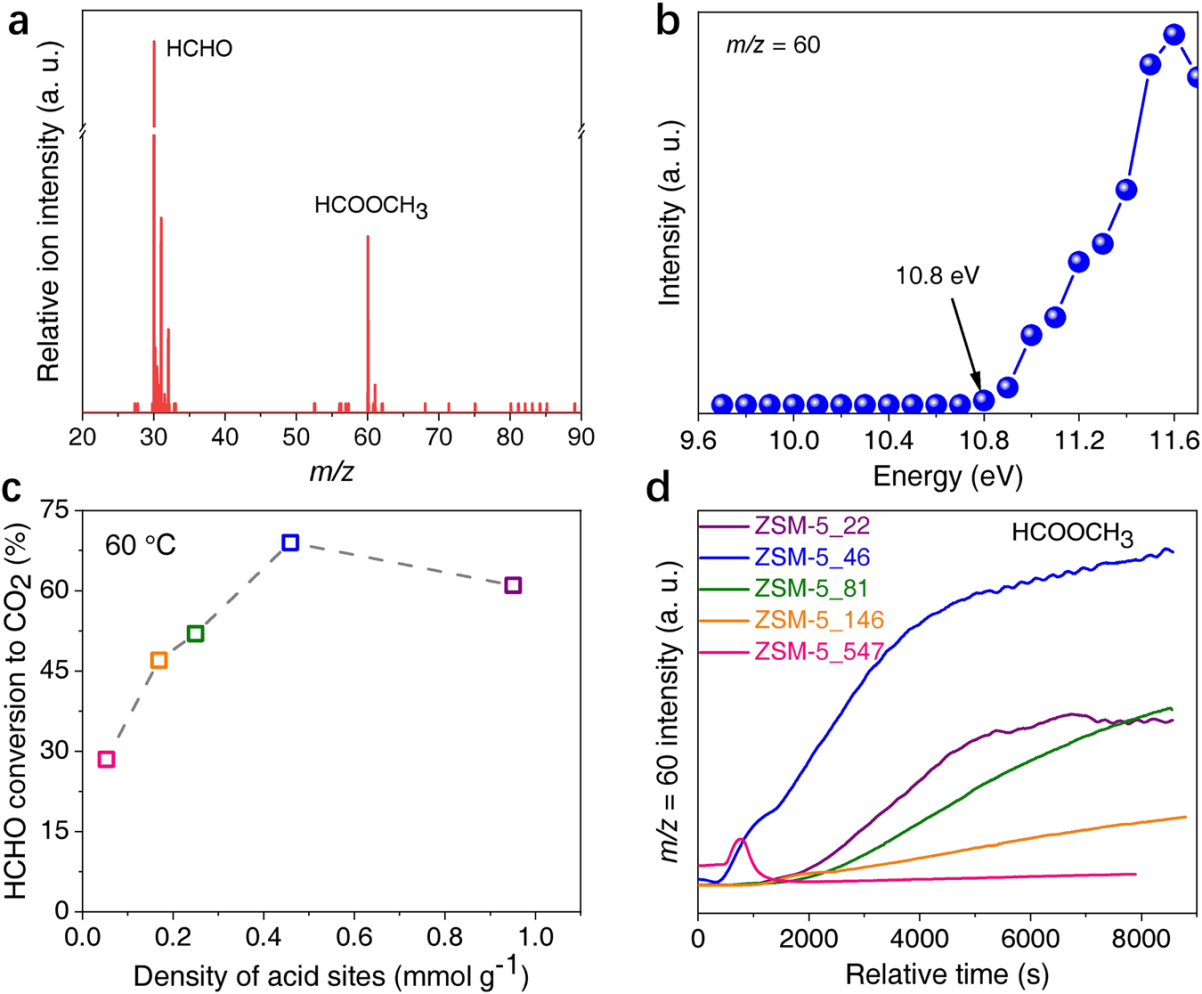
In situ investigation of gaseous intermediates and the role of ZSM-5 zeolite. **a** Study of HCHO conversion over ZSM-5 (SiO_2_/Al_2_O_3_ = 46) by SVUV-PIMS at the photon energy of 11.3 eV. **b** The PIE spectrum of *m*/*z* = 60 species in **a**. **c** Effect of acid density of ZSM-5 on HCHO conversion. The molar ratios of SiO_2_/Al_2_O_3_ from left to right are 547, 146, 81, 46, and 22, respectively. **d** Gaseous intermediate generation monitoring by an online quadrupole mass spectrometer. Activities of HCHO conversion to HCOOCH_3_ (*m*/*z* = 60) over ZSM-5 with different densities of acid sites at 60 °C.

To detect the gaseous intermediates, we turn to high sensitive synchrotron-based vacuum ultraviolet photoionization mass spectrometry (SVUV-PIMS), which is often used to detect gaseous intermediates or active radicals with trace amounts in heterogeneous catalysis and combustion fields^29^. In Fig. 2a, when HCHO carried by 20% O_2_–Ar is fed into the reactor in the presence of ZSM-5 (SiO_2_/Al_2_O_3_ = 46) alone at 65 °C, a distinct signal of *m*/*z* (mass/charge ratio) = 60 appears over time besides that of *m*/*z* = 30 for raw material of formaldehyde at a photon energy of 11.3 eV in the effluents. Due to the tunable photon energy, species can be distinguished exclusively based on the near-threshold ionization characteristic^10,29^. Scanning photoionization-efficiency (PIE) spectrum confirms that *m*/*z* = 60 signal can be unambiguously attributed to methyl formate (MF, HCOOCH_3_), whose distinguishable onset is 10.8 eV and the variation trend of ionization-energy (IE) intensity (Fig. 2b) is consistent with that of MF reported previously^30^. MF can be produced via formaldehyde disproportionation, which has been reported in previous research^31^. Density functional theory (DFT) calculations also show that the C=O of HCHO can be activated by hydrogen transfer from the Brønsted acid sites of ZSM-5, thus resulting in the weakening of C=O bonds, which eventually results in a new C=O bond formation in HCOOCH_3_ with a low barrier (Supplementary Fig. 7), consistent with the above in situ measurement shown in Fig. 2a.

Model experiments over ZSM-5 with different acidities (Supplementary Fig. 8) were further conducted to investigate the role of ZSM-5 component. In Fig. 2c, HCHO conversion demonstrates high dependence on the density of medium-strength acid sites of ZSM-5 zeolite when combined with Ag/SBA-15 component separately at 60 °C. ZSM-5_547 (SiO_2_/Al_2_O_3_ = 547) with an acid density of 0.053 mmol g^−1^ coupled with Ag/SBA-15 gives 29% HCHO conversion, while it is improved to 47% at a high density of 0.17 mmol g^−1^ of ZSM-5 (ZSM-5_146−Ag/SBA-15). Further improving acid amount to 0.46 mmol g^−1^, HCHO conversion is significantly advanced to 69% (ZSM-5_46−Ag/SBA-15), whereas it decreases to 61% at a higher amount of 0.95 mmol g^−1^ (ZSM-5_22−Ag/SBA-15). Therefore, appropriate acidity of ZSM-5 zeolite is essential to ensure the superior performance of HCHO oxidation. Correspondingly, HCHO activation over above ZSM-5 zeolites was also carried out with effluents monitored by an online quadrupole mass spectrometer at 60 °C (Fig. 2d). As expected, the detected *m*/*z* = 60 (MF) signals show an obvious dependence on acid density of ZSM-5 zeolite, and MF intensity follows the sequence of ZSM-5_46 > ZSM-5_22 > ZSM-5_81 > ZSM-5_146 > ZSM-5_547 (Fig. 2d). It is the same as that of the activity of the corresponding bifunctional ZSM-5−Ag/SBA-15 catalyst. The above experiments reveal the significant role of MF as a gaseous intermediate in HCHO oxidation.

Since ZSM-5 zeolite generally possesses multiple sites such as Brønsted acid sites, Lewis acid sites, and Si–OH, model experiments were performed to distinguish the crucial sites for HCHO activation. Silicalite-1 (denoted as S-1), a pure silica zeolite with the same skeleton structure of ZSM-5 (Supplementary Fig. 3) and most Si–OH sites at surfaces, and Al_2_O_3_ containing most Lewis acid sites, were investigated when combined with Ag/SBA-15 separately. Interestingly, S-1−Ag/SBA-15 and Al_2_O_3_−Ag/SBA-15 all give inferior activities with the complete conversion temperature above 145 °C (Supplementary Fig. 9), indicating the inappreciable role of the skeleton structure, Si–OH of zeolites, and Lewis acid sites in HCHO activation generating active intermediates. In addition, in situ SVUV-PIMS studies of HCHO activation over S-1 and Al_2_O_3_ (Supplementary Fig. 10) also confirm the above results, due to no *m*/*z* = 60 and other signals are detected, except for signals of raw materials (*m*/*z* = 30 for HCHO, *m*/*z* = 61 and 89 for 1,3,5-trioxane). Therefore, Brønsted acid sites over ZSM-5 seem to be responsible for the activation of HCHO to generate active intermediate of MF.

### Role of Ag in transformation of gaseous intermediates

**Fig. 3:**
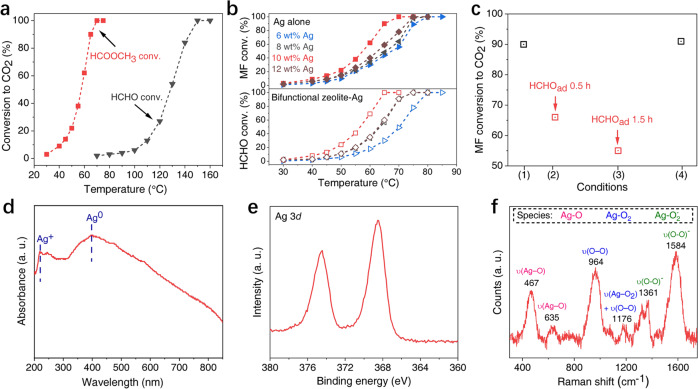
Role of Ag component in MF conversion. MF and HCHO conversion over (**a**) 10Ag/SBA-15 and **b** nAg/SBA-15 with different Ag loading. **c** Effect of HCHO pre-adsorption on MF oxidation over 10Ag/SBA-15. Condition 1: direct MF conversion without HCHO pre-adsorption; MF conversion after HCHO pre-adsorption for 0.5 h (condition 2) and 1.5 h (condition 3) at room temperature; Condition 4: after HCHO pre-adsorption for 1.5 h at room temperature, the catalyst was purged at 200 °C under flowing Ar for 0.5 h to remove adsorbed HCHO, then MF conversion was conducted. Reaction conditions: 65 °C, 50 ppm MF, and 36000 mL g^−1^ h^−^^1^. **d** UV–vis spectrum, **e** XPS spectrum of Ag 3*d*, and **f** Raman spectrum of 10Ag/SBA-15.

Subsequently, model experiments of MF conversion over Ag/SBA-15 component were studied. In Fig. 3a, the oxidation activity of MF (red square) is significantly superior to that of HCHO (gray triangle) over the same Ag/SBA-15 under the same reaction conditions, and their complete conversion temperatures are 65 °C and 150 °C, respectively. Furthermore, a series of Ag catalysts with different Ag loading (6, 8, 10, and 12 wt%) were tested. In Fig. 3b, they all exhibit excellent low-temperature activities of MF oxidation and the complete conversion temperatures are all below 80 °C (the upper figure). Interestingly, this superior activity follows the same sequence as that of the corresponding bifunctional ZSM-5−Ag/SBA-15 catalyst (the lower figure). For better visualization, MF conversion at 60 °C is displayed in Supplementary Fig. 11. The activity follows the order of 10Ag/SBA-15 > 12Ag/SBA-15 ~8Ag/SBA-15 > 6Ag/SBA-15, and 10Ag/SBA-15 gives the highest MF conversion of 62%, an approach to 69% HCHO conversion of the corresponding bifunctional ZSM-5−10Ag/SBA-15 catalyst. Therefore, MF as the key intermediate bridges ZSM-5 and Ag/SBA-15 components and enhances significantly the overall activity of this bifunctional catalyst via tandem process.

In addition, due to the presence of HCHO in the reaction atmosphere, we further investigated its influences on MF oxidation over Ag/SBA-15 component. To simplify, MF conversion over 10Ag/SBA-15 with and without HCHO pre-adsorption was conducted. As presented by Fig. 3c, compared with MF conversion over 10Ag/SBA-15 without HCHO pre-adsorption (black square, condition 1), HCHO pre-adsorption on Ag/SBA-15 can significantly reduce the activity of MF conversion (red square, condition 2) at the same reaction conditions, and the longer the pre-adsorption time of HCHO, the lower the MF conversion (red square, condition 3). More importantly, after removal of adsorbed HCHO by calcining the catalyst of “condition 3”, the activity of MF oxidation (black square, condition 4) can be recovered to the same conversion level of “condition 1”. This HCHO pre-adsorption phenomenon can well explain the suppression of overall activity of bifunctional catalysts when the upper component is insufficient (Fig. 1c) or the two components are packed too close (Fig. 1d). Because in both cases, the subsequent Ag components will inevitably adsorb unreacted HCHO molecules and thereby lead to lower MF oxidation activity.

Further, the state and role of Ag component were also investigated. Ultraviolet–visible (UV–vis) absorption spectrum shows two broad absorption bands at around 220 and 400 nm separately (Fig. 3d). The former one is assigned to the 4*d*^10^–4*d*^9^5*s*^1^ transition of Ag^+^ ions isolated on support surface, and the latter band corresponds to metallic Ag^0^ species^32^. X-ray photoelectron spectroscopy (XPS) spectrum of Ag 3*d* at 368.7 eV also confirms the major state of Ag^0^ on surfaces (Fig. 3e)^33,34^. Raman spectrum in Fig. 3f reveals the existence of different forms of chemisorbed oxygen species. Bands at 467 and 635 cm^−1^ are attributed to surface atomic oxygen species weakly chemisorbed on Ag(100) and Ag(111) planes with bridging Ag−O−Ag bonding, and subsurface atomic oxygen related to a υ(Ag–O) stretching vibration, respectively^34,35^. Those bands at 964 and 1176 cm^−1^ are associated with υ(O–O) vibration assigned to molecular oxygen species chemisorbed on defects^34–38^, and vibrations at 1361 and 1584 cm^−^^1^ are ascribed to the υ(O–O)^−^ mode of surface superoxidic species (nominally O_2_^−^)^38–40^. This indicates that Ag/SBA-15 can adsorb and activate O_2_, which may facilitate the oxidation process.

### In situ DRIFTS analysis of reaction mechanisms

In situ diffuse reflection infrared Fourier transform spectroscopy (DRIFTS) was measured to detect adsorbed surface intermediates after exposure of Ag/SBA-15 to MF and HCHO separately, and the subsequent oxidation processes were also investigated.

**Fig. 4:**
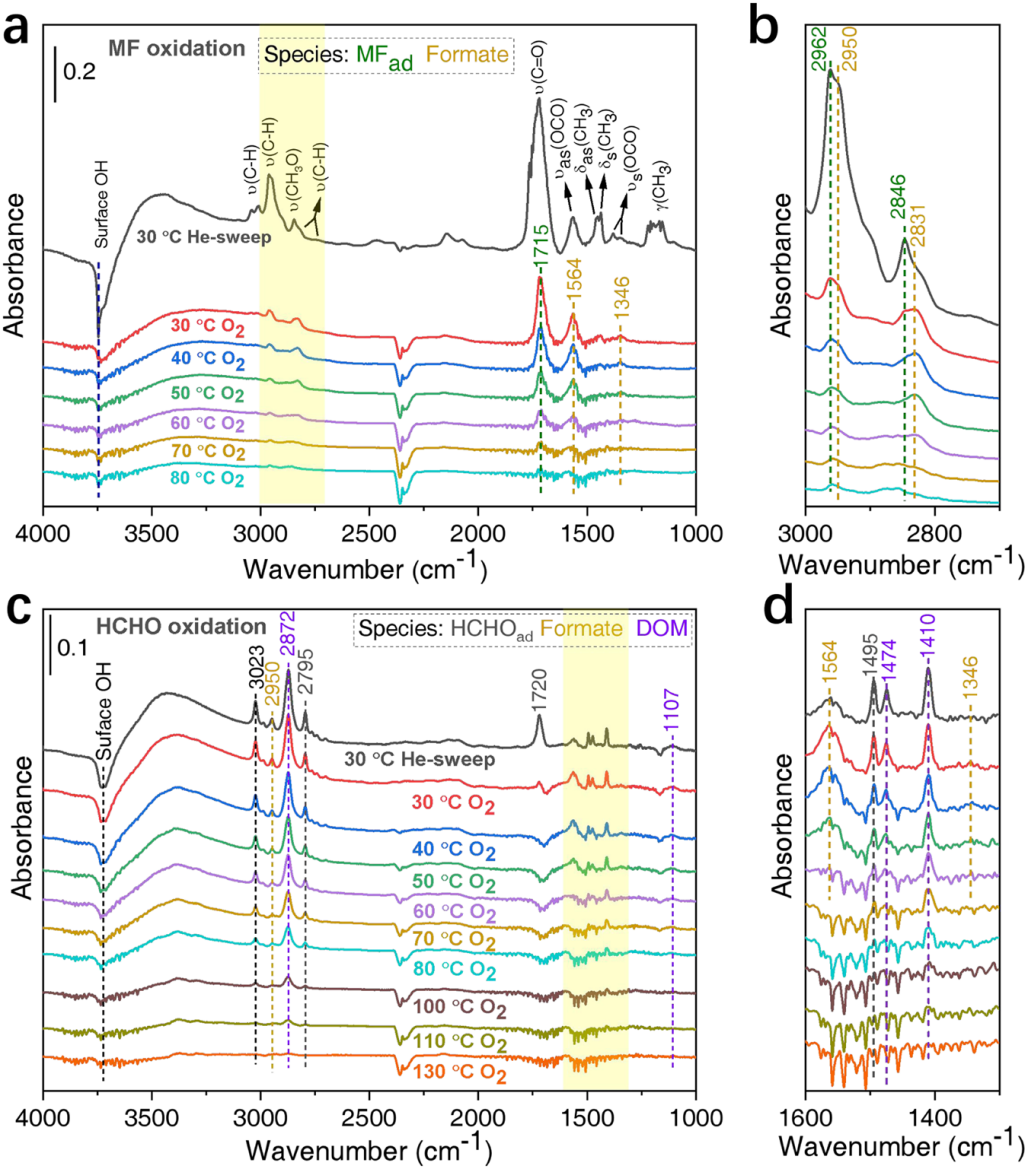
In situ DRIFT spectra of the oxidation processes. **a**, **b** MF and **c**, **d** HCHO oxidation over Ag/SBA-15 component, respectively. **b** and **d** are the enlarged images of the shaded parts in **a** and **c**, respectively. After adsorption of MF or HCHO for 10 min, Ag/SBA-15 was purged with helium, and then switched to 20%O_2_–He for a temperature-programmed reaction.

As Ag/SBA-15 is exposed to MF at 30 °C, an obvious reverse signal at 3700 cm^−1^ represented surface hydroxyls^41,42^ appearing, and its intensity increases gradually as time (Supplementary Fig. 12a), indicating the consumption of surface hydroxyls due to MF adsorption. Meanwhile, the typical vibrations of MF (bands at ~3500, 3080–2800, 2500–2300, 2200–2000, 1850–1650, 1452, and 1270–1070 cm^−1^, similar to the reference in Supplementary Fig. 12c) and formate (1564 and 1346 cm^−1^)^31,42,43^ species are also observed (the enlarged section in Supplementary Fig. 12a, Supplementary Table 1). The formation of formate species during MF adsorption at 30 °C indicates that Ag/SBA-15 component has a strong ability to activate MF at low temperatures. In Fig. 4a, b, after He sweeps at the same temperature, the signal intensity is reduced, but the wavenumber barely changes (gray line). However, upon switching to O_2_ at 30 °C, most of the characteristic vibrations of MF disappear, and only bands at 2962, 2846, and 1715 cm^−1^ for adsorbed MF^31,41,43,44^ remain, but their intensities are significantly reduced. In addition, signals of formate species are also apparent at 2950, 2831, 1564, and 1346 cm^−1^ (red line)^31,42,43,45–48^. As temperature increases to 40 °C, signals of adsorbed MF are obviously reduced than that of formate species, indicating that MF is easily converted at low temperatures. Further increasing temperatures from 50 to 70 °C, the intensities of adsorbed MF and formate species decrease significantly and disappear completely at 80 °C, accompanied by the gradual recovery of surface hydroxyls (3700 cm^−1^). CO_2_ (2359, 2341 cm^−^^1^)^41,49^ and H_2_O (1615 cm^−1^)^47^ are also formed during the oxidation process, which is consistent with the increased CO_2_ signals monitored by a mass spectrometer (Supplementary Fig. 13). Therefore, MF oxidation to CO_2_ over Ag/SBA-15 goes through adsorbed MF and formate species, both of which are easily oxidized to CO_2_ at low temperatures. It is also consistent with the catalytic activity in Fig. 3a–c.

Similarly, HCHO adsorption and oxidation were also conducted on Ag/SBA-15. After exposing to HCHO at 30 °C, surface hydroxyls (3700 cm^−1^) of Ag/SBA-15 are also consumed due to HCHO adsorption (Supplementary Fig. 12b, d). After He purification, the adsorbed species of most HCHO_ad_ (2795, 1720, and 1495 cm^−1^), dioxymethylene (DOM, 2872, 1474, 1410, and 1107 cm^−1^)^31,42,47,50–52^, and little formate (2950, 1564, and 1346 cm^−1^) are clearly observed in Fig. 4c, d (gray line). Then switching to O_2_ at the same temperature of 30 °C, the band at 1720 cm^−1^ of HCHO_ad_ decreases significantly, accompanied by the obvious increase of formate species at 1564 cm^−^^1^ (red line). Further increasing temperature from 40 to 60 °C, signals of formate species decrease significantly and almost disappear, which is similar to the observation in MF oxidation process, whereas the signal intensity of DOM decreases only a little. Thereafter, DOM remains dominant on surfaces and disappears completely at temperatures as high as 130 °C. Meanwhile, surface hydroxyls (3700 cm^−1^) are also gradually recovering and obvious CO_2_ is formed, which is consistent with the improved activity of HCHO oxidation on Ag/SBA-15 above 100 °C in Fig. 3a (gray line). This indicates that although the direct conversion of HCHO on Ag can also form easily converted formate species, there are still a large number of DOM species on surfaces, which requires higher temperatures to be completely converted. It may be the main reason for the low activity of HCHO oxidation directly on Ag/SBA-15. This mechanism is similar to that reported in previous studies based on L–H mechanism^53–55^, in which adsorbed HCHO can be oxidized on Ag catalyst via DOM and formate species, and then they are directly or indirectly converted to CO_2_ with most catalytic processes that take place at active Ag sites.

### DFT calculations of oxidation processes

DFT calculations were carried out for both MF and HCHO oxidation processes. Ag(111) was chosen as a representative for a close-packed surface and Ag(100) for an open facet. Calculated barriers (*G*_a_) and reaction energies (Δ*G*) of all considered elementary steps are listed in Supplementary Table 2. In addition, we have performed microkinetic modeling to analyze reaction rate, intermediates’ coverages, and degree of rate control, which is based on a steady-state approximation, described in the following and solved by CATKINAS^56,57^:

1 $$\frac{\partial {\theta }_{i}}{\partial t}=0$$

2 $$\mathop{\sum}\limits_{i}{\theta }_{i}=1$$

The reaction rate on surfaces was described by^58^

3 $$r={\theta }_{A}{\theta }_{B}\frac{{k}_{B}T}{h}{e}^{-{G}_{a}/{k}_{B}T}$$

Equations (1–3) were referred to a previous work^59^.

**Fig. 5:**
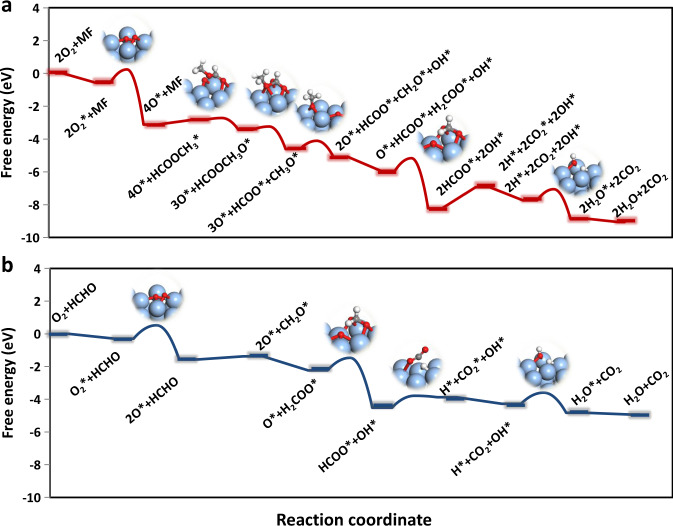
Free-energy diagrams of the oxidation processes. **a** MF (red line) and **b** HCHO (blue line) oxidation on Ag(100) surface, respectively. The transition-state structures are shown as insets, where the Ag, C, O, and H are represented in silver, gray, red, and white, respectively.

According to experimental conditions, the pressures are set for MF = 50 ppm, HCHO = 100 ppm, O_2_ = 0.2 bar, H_2_O = 100 ppm, and CO_2_ = 100 ppm. A critical reaction temperature of 333 K (60 °C) is chosen for analysis. Both formate and DOM routes are considered for MF and HCHO oxidation, respectively. The turnover frequencies (TOFs) are used to analyze the reaction rates. The TOFs of MF and HCHO oxidation on Ag(111) are low, 2.12 × 10^−7^ and 2.37 × 10^−7^, respectively (Supplementary Figs. 14 and 15). It is found that the O_2_ dissociation is the rate-determining step for both reactants’ oxidation. At the steady state, the surface is almost a free site (>90%) (#, Supplementary Fig. 16a, b). It indicates that a more reactive facet is needed for promoting reaction rate. Therefore, we analyzed the kinetics for MF and HCHO oxidation on an open Ag(100) facet. The TOFs of MF and HCHO oxidation on Ag(100) are 4.59 × 10^−4^ and 1.35 × 10^−^^5^, respectively (Supplementary Figs. 17 and 18), indeed higher in activity in comparison with Ag(111). According to the microkinetic results (Supplementary Figs. 14, 15, 17, and 18), the MF oxidation prefers to go through the following processes. First, it is oxidized to HCOOCH_3_O*, which subsequently dissociates into CH_3_O* and HCOO*. The CH_3_O* can be dehydrogenated to CH_2_O* with the assistance of O*. Then, it can be converted to H_2_COO*, which will be further dehydrogenated to HCOO* with the assistance of O*, and finally to CO_2_ (Fig. 5a and Supplementary Fig. 19a). In addition, the HCHO oxidation prefers the following steps with the first step directly converted to H_2_COO*. Then, it dissociates and produces HCOO* and finally to CO_2_, following an L–H mechanism (Fig. 5b and Supplementary Fig. 19b). The transition states of these critical steps are shown in Fig. 5 and the corresponding initial and final states over Ag(100) are shown in Supplementary Fig. 20.

In addition, the degree of (kinetic) rate control (*X*_RC_) was employed to analyze the importance and sensitivity of all elementary steps on the overall reaction rates. The O_2_* dissociation (*X*_RC_ = 0.83) and HCOO* dehydrogenation (*X*_RC_ = 0.16) are the top two of the most important elementary reactions for MF oxidation (Supplementary Table 3). In other words, it is beneficial for decreasing the barriers of the two steps to increase the activity of MF oxidation. By contrast, the H_2_COO* dehydrogenation (*X*_RC_ = 1.06), H_2_COO* formation (*X*_RC_ = −0.46), and O_2_* dissociation (*X*_RC_ = 0.37) are the most important elementary reactions for HCHO oxidation (Supplementary Table 4). Although the O_2_* dissociation was easier on Ag(100) compared with Ag(111), the H_2_COO* dehydrogenation became harder (0.49–0.77 eV), which needs the assistance of O*. In other words, a (slightly) larger barrier of H_2_COO* formation is beneficial to enhance the activity of HCHO oxidation. These are explained by the Sabatier principle that the interactions between the catalyst and the intermediates should be “just right”; if the binding is too weak, O_2_ will fail to bind to the catalyst and the oxidation is hard to happen. On the other hand, if the binding is too strong, the catalyst will be blocked by H_2_COO*, which is difficult to be converted. The H_2_COO* can be formed easily by HCHO direct oxidation, but the H_2_COO* is formed by several processes for MF oxidation, which can balance the formation and consumption of H_2_COO*. These can also explain the activity of MF conversion over Ag/SBA-15 with HCHO pre-adsorption, which is lower than that without HCHO pre-adsorption (Fig. 3c). The HCHO pre-adsorption can form excess H_2_COO*, which results in decreased activity. According to the microkinetic modeling at the steady state, for MF oxidation on Ag(100), the O_2_* coverage takes up 91.27% of the total sites, OH* and HCOO* take up 3.97% and 3.96%, respectively (Supplementary Fig. 16c). By contrast, for HCHO oxidation on Ag(100), the H_2_COO* and O_2_* take up 82.96% and 15.68% in total coverage, respectively (Supplementary Fig. 16d). It can also explain well the observation of formate (1564 and 1346 cm^−1^) species (Fig. 4a, b) and DOM (2872, 1474, 1410, and 1107 cm^−1^) species (Fig. 4c, d) in the in situ DRIFT spectra.

Overall, both experimental observations and theoretical calculations indicate that the MF oxidation is easier compared with HCHO oxidation over supported Ag catalyst. Hence, the bifunctional and tandem ZSM-5−Ag catalyst through MF intermediates is superior to the direct oxidation of HCHO over supported silver catalysts.

## Discussion

In summary, a new design strategy of bifunctional ZSM-5−Ag catalytic process is proposed for HCHO oxidation to CO_2_. It decreases the complete conversion temperature by nearly 85 °C compared with Ag component alone, and the complete conversion temperature can be achieved at 65 °C. This is enabled by the tandem catalytic process of bifunctional catalyst. First, HCHO is converted to MF over acidic ZSM-5 zeolite. Then, MF is transferred to the subsequent Ag component through gas phase and is further oxidized to CO_2_ with a higher TOF compared with HCHO oxidation. The cooperation of acidic ZSM-5 zeolite and Ag significantly increases the low-temperature activity of HCHO oxidation. This catalytic process is significantly different from that reported in previous studies, in which HCHO is directly oxidized via adsorbed DOM and formate species on supported Ag catalyst based on the L–H mechanism. Unfortunately, the DOM species generated here needs a higher temperature (130 °C) to be fully transformed, which limits its low-temperature activity. DFT calculations, in situ DRIFTS, in situ SVUV-PIMS, and model experiments confirmed the key intermediates of MF, and the superiority of MF oxidation than HCHO direct oxidation over Ag catalyst. These findings here open up a new avenue for the development of HCHO oxidation technologies, which may also give guidance for the oxidation of other VOCs in a more moderate way and provide prospects for the industrial application of low-cost catalysts.

## Methods

### Catalyst preparation

Commercial ZSM-5 zeolites were purchased from Nankai University Company. ZSM-5 zeolites with different molar ratio of SiO_2_/Al_2_O_3_ = 22, 46, 81, 146, and 547 were denoted as ZSM-5_22, ZSM-5_46, ZSM-5_81, ZSM-5_146, and ZSM-5_547, respectively. Note, ZSM-5 in bifunctional ZSM-5−Ag/SBA-15 catalyst generally refers to ZSM-5_46, unless otherwise stated.

Mesoporous SBA-15 sample was synthesized under acidic conditions using ethyl orthosilicate (TEOS) as silicon source and triblock copolymer (P123) as template^60^. The specific synthesis steps are as follows: 6 g of P123 was dissolved into a mixed solution of 45 mL of deionized water and 180 mL of hydrochloric acid (2 M) at 40 °C. Then, 12.75 g of TEOS was added to the above solution and stirred for 24 h. The resulting suspension was transferred into a Teflon-lined stainless-steel autoclave and heated at 100 °C for 48 h. The precipitate was filtered, washed with distilled water, and dried at 70 °C overnight. The white SBA-15 powder was obtained by calcining the precipitate in air at 550 °C with a heating rate of 1 °C/min. It was impregnated with an aqueous solution of a certain amount of silver nitrate, which was followed by stirring evenly and then quiescence for 24 h at room temperature. Then, the sample was dried at 80 °C for 24 h. Before reaction, the sample was calcinated in 20%O_2_−Ar at 600 °C for 2 h and then activated by heating in 10% H_2_–Ar at 50 °C for 1 h. The catalyst obtained was named as nAg/SBA-15, where “n” represented Ag loading of 6, 8, 10, and 12 in the unit of wt%, according to the ratio of Ag to SBA-15 support. Note, Ag/SBA-15 generally refers to 10Ag/SBA-15 in bifunctional zeolite−Ag catalyst, unless otherwise stated.

Zeolite and Ag/SBA-15 were used with 40−60 mesh (0.30−0.45 mm) particles, unless otherwise stated.

### Catalyst characterization

X-ray diffraction (XRD) was measured on a D/MAX 2500/PC equipped with a Cu K_α_ radiation source (*λ* = 1.5418 Å), operated at 200 mA and 40 kV. XRD pattern was recorded in the range of 2 theta = 10−90°. The crystal size of Ag crystals was estimated using the Scherrer equation. Transmission electron microscopy (TEM) images were obtained using an HT7700 electron microscope operated at an accelerating voltage of 100 kV. X-ray photoelectron spectroscopy (XPS) was measured on a Thermos K-Alpha+ spectrometer using an Al K_α_ (hν = 1486.6 eV, 1 eV = 1.603 × 10^−19^ J) X-ray source. The C 1 *s* binding energy at 284.8 eV was used for calibration. Ultraviolet–visible (UV–vis) diffuse reflectance spectrum was collected using a SHIMADZU UV-2600 spectrophotometer. Raman spectrum was recorded on a Renishaw PLC inVia Qontor spectrometer at room temperature with the excitation wavelength of 532 nm. Raman spectrum was obtained with automatic baseline correction. Temperature-programmed desorption of NH_3_ (NH_3_‒TPD) was performed on a Micromeritics AutoChem 2910 instrument equipped with a thermal conductivity detector (TCD). Typically, 100 mg of zeolites were loaded and pretreated under flowing Ar at 450 °C for 1.5 h. After cooling down to 100 °C under flowing Ar, the sample was exposed to 5% NH_3_–He at 100 °C. Then, the sample was swept by Ar at the same temperature till a stable baseline was obtained. Subsequently, the signal was recorded while the temperature was increased from 100 to 800 °C at a heating rate of 10 °C/min. In situ diffuse reflection infrared Fourier transform spectroscopy (DRIFTS) was measured on a Thermos Nicolet iS 10 equipped with an MCT detector at a spectral resolution of 4 cm^−1^ and an accumulation of 32 scans. Before the test, the sample was pretreated at 150 °C for 0.5 h in 20% O_2_–He. Background spectra were recorded at 130, 110, 100, 90, 80, 70, 60, 50, 40, and 30 °C in 20% O_2_–He, and 30 °C in He separately. Methyl formate or formaldehyde was fed into the sample cell at 30 °C for 10 min, and then purged by He. Thereafter, 20% O_2_–He was introduced into the sample cell at the same temperature. Then, the sample was heated up from 30 to 130 °C at a temperature interval of 10 °C in 20% O_2_–He. Sample spectra were obtained with the corresponding background spectra subtracted during the entire process.

### Catalytic reaction tests

Catalytic reactions were performed in a continuous-flow, fixed-bed reactor equipped with a quartz tube. Generally, 100 mg of catalyst (40−60 mesh) was used, in which the mass ratio of zeolite to Ag/SBA-15 is 1/4, and Ag/SBA-15 was packed below ZSM-5, separated by an inert layer of quartz wool at a distance of 3 mm, unless otherwise stated. Gas passes through 1,3,5-trioxane to bring formaldehyde into the reactor. The reaction was carried out under conditions of atmospheric pressure, 30–160 °C, 100 ppm HCHO, 20% O_2_–Ar, and gas hourly space velocity (GHSV) = 36000 mL g^−1^ h^−1^ unless otherwise stated.

Kinetic experiments were carried out under conditions of atmospheric pressure, 50–140 °C, 400 ppm HCHO, 20 mg of composite with the mass ratio of zeolite to Ag/SBA-15 is 1/4, and GHSV = 180000 mL g^−1^ h^−1^. The apparent activation energy (*E*_a_) was calculated based on the results of kinetic experiments with HCHO conversion below 15%.

Model experiments of methyl formate oxidation to CO_2_ and H_2_O were carried out on the same fixed-bed reactor as for HCHO conversion. Analogously, 100 mg of Ag/SBA-15 catalyst was used. The reaction was carried out under conditions of atmospheric pressure, 30−85 °C, 50 ppm HCOOCH_3_, 20% O_2_–Ar, and GHSV = 36000 mL g^−1^ h^−1^.

CO_2_ product was transformed to methane through a nickel catalyst converter under excess hydrogen. Methane was then analyzed by an online gas chromatography (GC 7890II, Tech comp, China) equipped with a flame ionization detector (FID).

HCHO conversion (Conv_HCHO_) was calculated on a carbon atom basis, i.e.,

4 $${{{{{{\rm{Conv}}}}}}}_{{{{{{\rm{HCHO}}}}}}}=\frac{{{{{{{\rm{HCHO}}}}}}}_{{{{{{\rm{inlet}}}}}}}-{{{{{{\rm{HCHO}}}}}}}_{{{{{{\rm{outlet}}}}}}}}{{{{{{{\rm{HCHO}}}}}}}_{{{{{{\rm{inlet}}}}}}}}\times 100 \%$$

where HCHO_inlet_ and HCHO_outlet_ in Eq. (4) represented moles of HCHO at the inlet and outlet, respectively.

Model experiments of HCHO conversion over ZSM-5, and methyl formate as well as HCHO conversion over Ag/SBA-15, were all carried out on another fixed-bed reactor equipped with an OmniStar 320 mass spectrometer (MS) detector. Typically, 70 mg of zeolite was used in HCHO conversion over ZSM-5 with different densities of acid sites at a constant temperature of 60 °C. Before tests, zeolites were pretreated at 500 °C for 1 h in 20% O_2_–He. Reaction conditions: atmospheric pressure and GHSV = 25714 mL g^−1^ h^−1^. About 50 mg of Ag/SBA-15 was used in methyl formate and HCHO conversion. Before the test, Ag/SBA-15 was pretreated at 200 °C for 0.5 h in 20% O_2_–He. The sample was measured at several settled temperatures in the range of 25–75 °C with each temperature maintained for 1 h. Activity data of CO_2_ (*m*/*z* = 44) formation were collected at 0.5 h for each settled temperature.

Model experiments of HCHO conversion over ZSM-5, S-1, and Al_2_O_3_ (Fig. 2a, b, and Supplementary Fig. 10) were carried out at the same mass spectrometry end station of the National Synchrotron Radiation Laboratory (NSRL) in Hefei, China. The experimental setup consists of a bubbler feeding system, a low-pressure catalytic reactor, and an orthogonal time-of-flight mass spectrometer (TOF-MS) using synchrotron vacuum ultraviolet (VUV) light with the photon energy continuously adjustable from 7.5 to 22 eV as the ionization source. About 20%O_2_–Ar carried HCHO in ice bath. Reaction conditions: 2 torr, 50 mg of catalyst, 65 °C, and GHSV = 21000 mL g^−1^ h^−1^.

### Computational details

We have employed the Vienna Ab initio Simulation Package (VASP) to perform all density functional theory (DFT)^61,62^ calculations with the generalized gradient approximation (GGA) using the revised Perdew–Burke–Ernzerhof (rPBE)^63^ function. We have chosen the projected augmented wave (PAW)^64,65^ potentials to describe the ionic cores and take valence electrons into account using a plane-wave basis set with a kinetic energy cutoff of 450 eV. Geometry optimizations were performed with the force convergency smaller than 0.05 eV/Å. The van der Waals (vdW) corrections have been introduced^66^. The reaction free energies (Δ*G*) were calculated as follows: Δ*G* = Δ*E* + Δ*ZPE* – *T*Δ*S* (*T* = 333 K). Where Δ*E* is the electronic energy based on DFT calculations directly, Δ*ZPE* and Δ*S* are the corrections of zero-point energy and entropy. Only vibrational motion was considered for adsorbates on the surface, while translational, rotational, and vibrational motions were all calculated for gas-phase species. The climbing-image nudged elastic-band (CI-NEB) method was used to locate the transition states^67^.

### Model construction

The ZSM-5 zeolite structure was modeled with a 14-T cluster (Si_13_AlO_14_H_29_). The cluster was generated from MFI crystal structure and the edge of the cluster was saturated by H atoms. The terminal H atoms were fixed, while the other framework atoms were allowed to relax. The four-layer (4 × 4) Ag(111) and (2 × 2) Ag(100) were built. The two layers at the bottom were fixed, while the other atoms relaxed. Monkhorst–Pack k-points of (1 × 1 × 1), (2 × 2 × 1), and (3 × 3 × 1) were applied for all the surface calculations for ZSM-5, Ag(111), and Ag(100), respectively.

## Supplementary information

Supplementary Information

## Data Availability

All other data supporting the findings are available in the article as well as the supplementary information files and can also be found from the authors on reasonable request.

## Code availability

The code used to generate the data shown in the main text is available from the corresponding author upon request.

## References

[1] Spivey JJ (1987). Complete catalytic oxidation of volatile organics. Ind. Eng. Chem. Res..

[2] Guo Y, Wen M, Li G, An T (2021). Recent advances in VOC elimination by catalytic oxidation technology onto various nanoparticles catalysts: a critical review. Appl. Catal. B Environ..

[3] Tang XF (2006). MnO_x_-CeO_2_ mixed oxide catalysts for complete oxidation of formaldehyde: effect of preparation method and calcination temperature. Appl. Catal. B Environ..

[4] Zhang C, He H, Tanaka K-I (2006). Catalytic performance and mechanism of a Pt/TiO_2_ catalyst for the oxidation of formaldehyde at room temperature. Appl. Catal. B Environ..

[5] Huang H, Xu Y, Feng Q, Leung DYC (2015). Low temperature catalytic oxidation of volatile organic compounds: a review. Catal. Sci. Technol..

[6] Tang X, Chen J, Huang X, Xu Y, Shen W (2008). Pt/MnO_x_-CeO_2_ catalysts for the complete oxidation of formaldehyde at ambient temperature. Appl. Catal. B Environ..

[7] Nie LH (2013). Enhanced performance of NaOH-modified Pt/TiO_2_ toward room temperature selective oxidation of formaldehyde. Environ. Sci. Technol..

[8] Zhang J, Li Y, Wang L, Zhang C, He H (2015). Catalytic oxidation of formaldehyde over manganese oxides with different crystal structures. Catal. Sci. Technol..

[9] Huang Z (2012). Catalytically active single-atom sites fabricated from silver particles. Angew. Chem. Int. Ed..

[10] Jiao F (2016). Selective conversion of syngas to light olefins. Science.

[11] Gao P (2017). Direct conversion of CO_2_ into liquid fuels with high selectivity over a bifunctional catalyst. Nat. Chem..

[12] Li N (2019). High-quality gasoline directly from syngas by dual metal oxide-zeolite (OX-ZEO). Catal. Angew. Chem. Int. Ed..

[13] Kang J (2020). Single-pass transformation of syngas into ethanol with high selectivity by triple tandem catalysis. Nat. Commun..

[14] Yamada Y (2011). Nanocrystal bilayer for tandem catalysis. Nat. Chem..

[15] Wei J (2017). Directly converting CO_2_ into a gasoline fuel. Nat. Commun..

[16] Cheng K (2017). Bifunctional catalysts for one-step conversion of syngas into aromatics with excellent selectivity and stability. Chem.

[17] Li Z (2019). Highly selective conversion of carbon dioxide to aromatics over tandem catalysts. Joule.

[18] Wei J, Yao R, Han Y, Ge Q, Sun J (2021). Towards the development of the emerging process of CO_2_ heterogenous hydrogenation into high-value unsaturated heavy hydrocarbons. Chem. Soc. Rev..

[19] Zecevic J, Vanbutsele G, de Jong KP, Martens JA (2015). Nanoscale intimacy in bifunctional catalysts for selective conversion of hydrocarbons. Nature.

[20] Zhou W (2018). Direct conversion of syngas into methyl acetate, ethanol, and ethylene by relay catalysis via the intermediate dimethyl ether. Angew. Chem. Int. Ed..

[21] Li N (2019). Size effects of ZnO nanoparticles in bifunctional catalysts for selective syngas conversion. ACS Catal..

[22] Ding Y (2021). Effects of proximity-dependent metal migration on bifunctional composites catalyzed syngas to olefins. ACS Catal..

[23] Qu Z, Shen S, Chen D, Wang Y (2012). Highly active Ag/SBA-15 catalyst using post-grafting method for formaldehyde oxidation. J. Mol. Catal. A Chem..

[24] Chen D, Qu Z, Sun Y, Gao K, Wang Y (2013). Identification of reaction intermediates and mechanism responsible for highly active HCHO oxidation on Ag/MCM-41. catalysts. Appl. Catal. B Environ..

[25] Bai B, Li J (2014). Positive effects of K^+^ Ions on three-dimensional mesoporous Ag/Co_3_O_4_ catalyst for HCHO oxidation. ACS Catal..

[26] Ma L (2014). Ag/CeO_2_ nanospheres: Efficient catalysts for formaldehyde oxidation. Appl. Catal. B Environ..

[27] Li J (2018). Integrated tuneable synthesis of liquid fuels via Fischer-Tropsch technology. Nat. Catal..

[28] Weisz PB (1962). Polyfunctional heterogeneous catalysis. Adv. Catal..

[29] Wen W (2020). Formation and fate of formaldehyde in methanol-to-hydrocarbon reaction: in situ synchrotron radiation photoionization mass spectrometry study. Angew. Chem. Int. Ed..

[30] Wang J, Yang B, Cool TA, Hansen N (2010). Absolute cross-sections for dissociative photoionization of some small esters. Int. J. Mass Spectrom..

[31] Popova GY, Andrushkevich TV, Chesalov YA, Stoyanov ES (2000). In situ FTIR study of the adsorption of formaldehyde, formic acid, and methyl formiate at the surface of TiO_2_ (anatase). Kinet. Catal..

[32] Chen X (2018). Specific role of potassium in promoting Ag/Al_2_O_3_ for catalytic oxidation of formaldehyde at low temperature. J. Phys. Chem. C.

[33] Tang X (2006). Complete oxidation of formaldehyde over Ag/MnO_x_-CeO_2_ catalysts. Chem. Eng. J..

[34] Zhao Z, Carpenter MA (2013). Support-free bimodal distribution of plasmonically active Ag/AgO_x_ nanoparticle catalysts: attributes and plasmon enhanced surface chemistry. J. Phys. Chem. C.

[35] Waterhouse GIN, Bowmaker GA, Metson JB (2003). Oxygen chemisorption on an electrolytic silver catalyst: a combined TPD and Raman spectroscopic study. Appl. Surf. Sci..

[36] Wang XD, Greenler RG (1991). Direct structure determination of oxygen adsorbed on silver by reflection-absorption infrared spectroscopy with isotopic substitution. Phys. Rev. B: Condens. Matter.

[37] Gang L, Anderson BG, van Grondelle J, van Santen RA (2003). Low temperature selective oxidation of ammonia to nitrogen on silver-based catalysts. Appl. Catal. B Environ..

[38] Ren L (2006). Transformation of various oxygen species on the surface of electrolytic silver characterized by in situ Raman spectroscopy. Chin. J. Catal..

[39] Prabhakaran K, Rao CNR (1987). A combined EELS-XPS study of molecularly chemisorbed oxygen on silver surfaces: evidence for superoxo and peroxo species. Surf. Sci..

[40] Kondarides DI, Papatheodorou GN, Vayenas CG, Verykios XE (1993). In situ high temperature SERS study of oxygen adsorbed on Ag: support and electrochemical promotion effects. Ber. Bunsenges. Phys. Chem..

[41] Chuang CC, Wu WC, Huang MC, Huang IC, Lin JL (1999). FTIR study of adsorption and reactions of methyl formate on powdered TiO_2_. J. Catal..

[42] Zhu X, Yu J, Jiang C, Cheng B (2017). Catalytic decomposition and mechanism of formaldehyde over Pt-Al_2_O_3_ molecular sieves at room temperature. Phys. Chem. Chem. Phys..

[43] Millar GJ, Rochester CH, Waugh KC (1991). Infrared study of methyl formate and formaldehyde adsorption on reduced and oxidised silica-supported copper catalysts. J. Chem. Soc. Faraday Trans..

[44] Gazsi A, Schubert G, Pusztai P, Solymosi F (2013). Photocatalytic decomposition of formic acid and methyl formate on TiO_2_ doped with N and promoted with Au. Production of H_2_. Int. J. Hydrog. Energ..

[45] Millar GJ, Metson JB, Bowmaker GA, Cooney RP (1994). An in situ Fourier transform Infrared study of formic acid adsorption on a polycrystalline silver catalyst. J. Catal..

[46] Millar GJ, Metson JB, Bowmaker GA, Cooney RP (1995). Influence of oxidation and reduction conditions upon the morphology of silica-supported polycrystalline silver catalysts. J. Chem. Soc. Faraday Trans..

[47] Shi C (2012). Catalytic formaldehyde removal by “storage-oxidation” cycling process over supported silver catalysts. Chem. Eng. J..

[48] Ou CC, Chen CH, Chan TS, Chen CS, Cheng S (2019). Influence of pretreatment on the catalytic performance of Ag/CeO_2_ for formaldehyde removal at low temperature. J. Catal..

[49] Imamura S, Uchihori D, Utani K, Ito T (1994). Oxidative decomposition of formaldehyde on silver-cerium composite oxide catalyst. Catal. Lett..

[50] Millar GJ, Rochester CH, Waugh KC (1995). An FTIR study of the adsorption of formic acid and formaldehyde on potassium-promoted Cu/SiO_2_ catalysts. J. Catal..

[51] Rasko J, Kecskes T, Kiss J (2004). Adsorption and reaction of formaldehyde on TiO_2_-supported Rh catalysts studied by FTIR and mass spectrometry. J. Catal..

[52] Xu B, Zhu T, Tang X, Shang J (2010). Heterogeneous reaction of formaldehyde on the surface of TiO_2_ particles. SCI China Chem..

[53] Wu H, Ma S, Song W, Hensen EJM (2016). Density functional theory study of the mechanism of formaldehyde oxidation on Mn-doped ceria. J. Phys. Chem. C.

[54] Ding J, Yang Y, Liu J, Wang Z (2020). Catalytic reaction mechanism of formaldehyde oxidation by oxygen species over Pt/TiO_2_ catalyst. Chemosphere.

[55] Wang Y (2020). Unveiling the effects of alkali metal ions intercalated in layered MnO_2_ for formaldehyde catalytic oxidation. ACS Catal..

[56] Chen J, Jia M, Hu P, Wang H (2021). CATKINAS: A large-scale catalytic microkinetic analysis software for mechanism auto-analysis and catalyst screening. J. Comput. Chem..

[57] Guo C, Mao Y, Yao Z, Chen J, Hu P (2019). Examination of the key issues in microkinetics: CO oxidation on Rh(111). J. Catal..

[58] Chorkendorff, I. & Niemantsverdriet, J. W. *Concepts of Modern Catalysis and Kinetics*. 79–128 (John Wiley & Sons, 2003).

[59] Fu X, Li J, Long J, Guo C, Xiao J (2021). Understanding the product selectivity of syngas conversion on ZnO surfaces with complex reaction network and structural evolution. ACS Catal..

[60] Zhao DY (1998). Triblock copolymer syntheses of mesoporous silica with periodic 50 to 300 angstrom pores. Science.

[61] Kresse G, Hafner J (1994). Ab initio molecular-dynamics simulation of the liquid-metal–amorphous-semiconductor transition in germanium. Phys. Rev. B.

[62] Kresse G, Furthmuller J (1996). Efficiency of ab-initio total energy calculations for metals and semiconductors using a plane-wave basis set. Comput. Mater. Sci..

[63] Perdew JP, Burke K, Ernzerhof M (1996). Generalized gradient approximation made simple. Phys. Rev. Lett..

[64] Blochl PE, Jepsen O, Andersen OK (1994). Improved tetrahedron method for Brillouin-zone integrations. Phys. Rev. B.

[65] Kresse G, Joubert D (1999). From ultrasoft pseudopotentials to the projector augmented-wave method. Phys. Rev. B.

[66] Grimme S, Antony J, Ehrlich S, Krieg H (2010). A consistent and accurate ab initio parametrization of density functional dispersion correction (DFT-D) for the 94 elements H-Pu. J. Chem. Phys..

[67] Henkelman G, Uberuaga BP, Jonsson H (2000). A climbing image nudged elastic band method for finding saddle points and minimum energy paths. J. Chem. Phys..

