# Supplementary material for: Bifunctional zeolites-silver catalyst enabled tandem oxidation of formaldehyde at low temperatures
Source: Nat Commun. 2022 Apr 22;13:2209. doi: 10.1038/s41467-022-29936-8 (PMC9033842; doi:10.1038/s41467-022-29936-8)
Supplement: Supplementary file 1 — Supplementary Information [file 41467_2022_29936_MOESM1_ESM.pdf]

Supplementary Information

Bifunctional Zeolites-Silver catalyst enabled tandem oxidation of formaldehyde at low temperatures

Na Li[1,†], Bin Huang[1,†], Xue Dong[2,†], Jinsong Luo[3], Yi Wang[1], Hui Wang[1], Dengyun Miao[2], Yang Pan[3], Feng Jiao[2], Jianping Xiao[2*] and Zhenping Qu[1*]*

[1] Key Laboratory of Industrial Ecology and Environmental Engineering (Ministry of Education, China), School of Environmental Science and Technology, Dalian University of Technology, Linggong Road 2, Dalian 116024, China

[2] Dalian Institute of Chemical Physics, Chinese Academy of Sciences, 457 Zhongshan Road, Dalian 116023, China

[3] National Synchrotron Radiation Laboratory, University of Science and Technology of China, Jinzhai Road 96, Hefei 230026, China

[†] These authors contributed equally: Na Li, Bin Huang, and Xue Dong.

email: jiaofeng@dicp.ac.cn; xiao@dicp.ac.cn; quzhenping@dlut.edu.cn

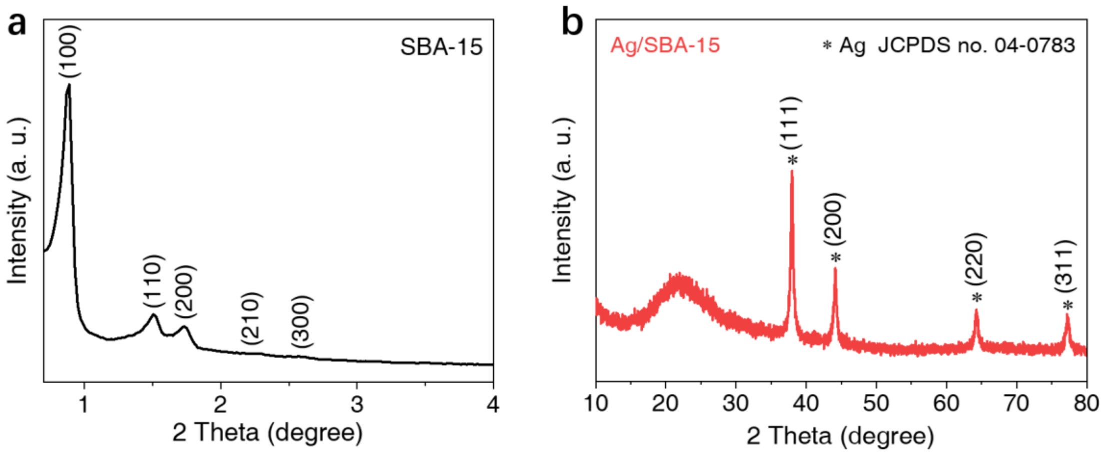

Supplementary Figure 1. XRD patterns of silver-related components. a SBA-15. **b** Ag/SBA-15.

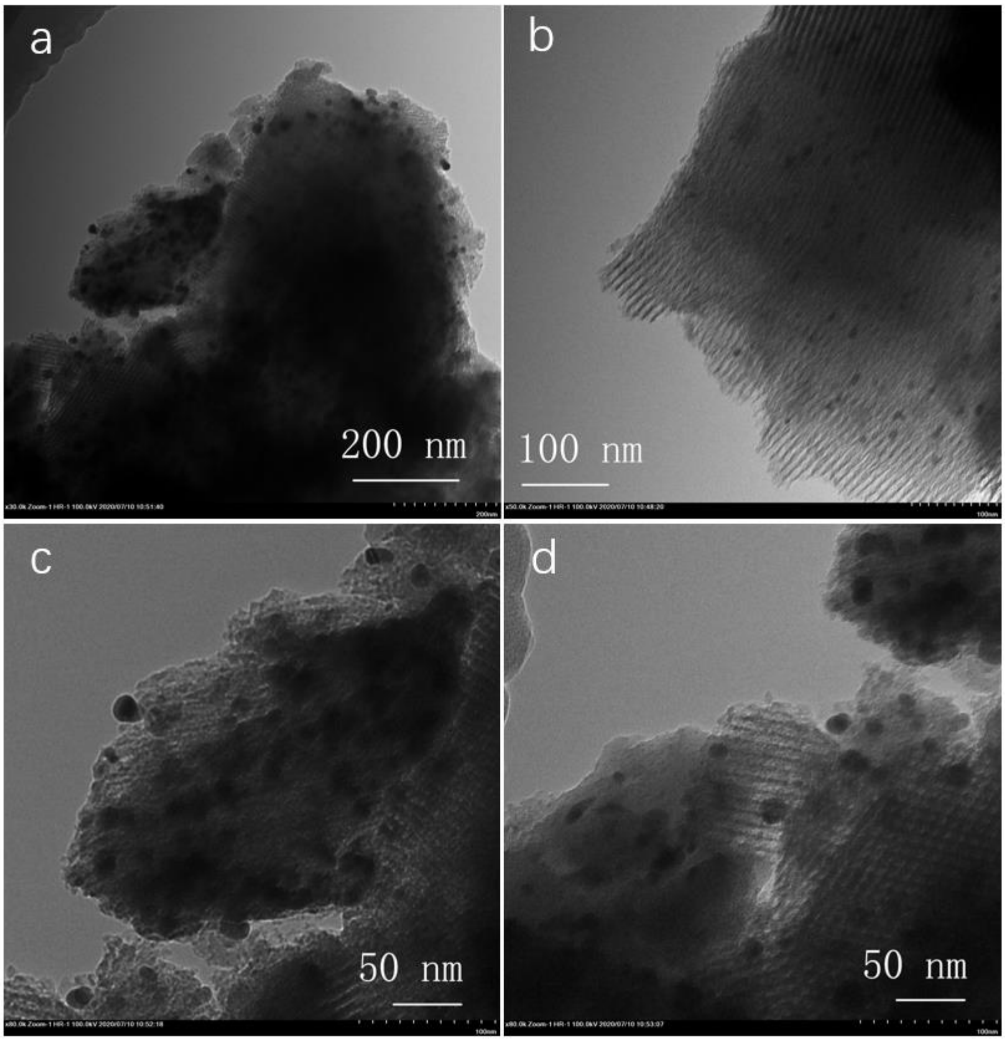

Supplementary Figure 2. TEM images of Ag/SBA-15. In **a-d** images, SBA-15 contains regular mesoporous channels, and Ag exists in the form of nanoparticles.

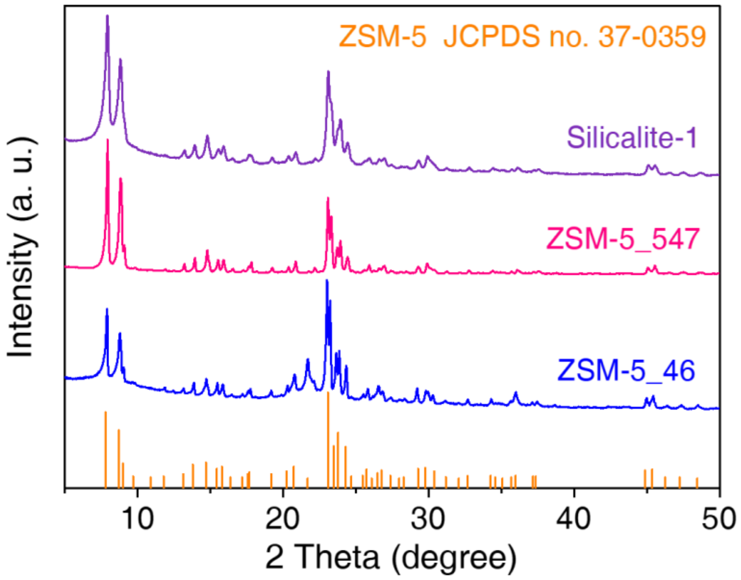

Supplementary Figure 3. XRD patterns of zeolites. ZSM-5 with different SiO_2/Al_2O_3 ratios.

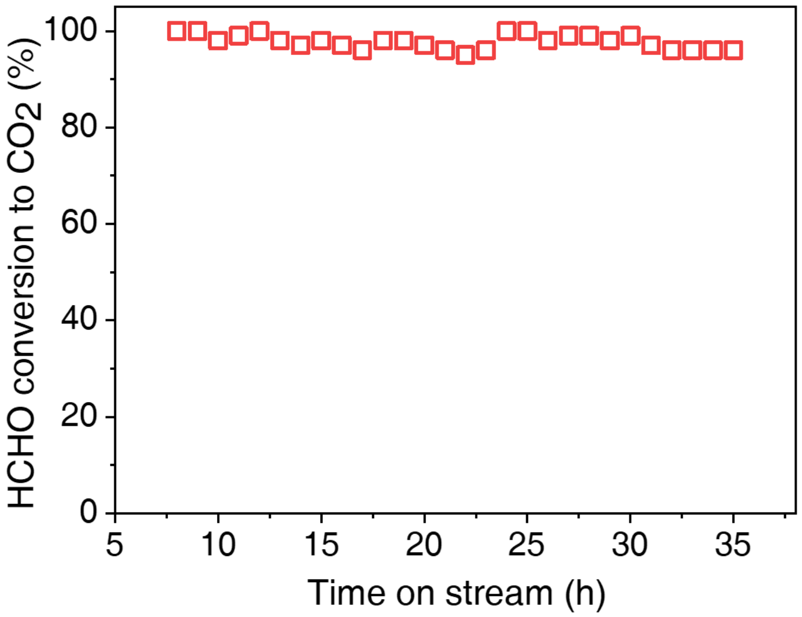

Supplementary Figure 4. Stability test of bifunctional ZSM-5–Ag/SBA-15 catalyst. Reaction conditions: the mass ratio of ZSM-5_46 to 10Ag/SBA-15 is 1/4, 100 ppm HCHO, 20% O_2-Ar, 70 ^{o}C, GHSV = 36000 mL g^{-1} h^{-1}.

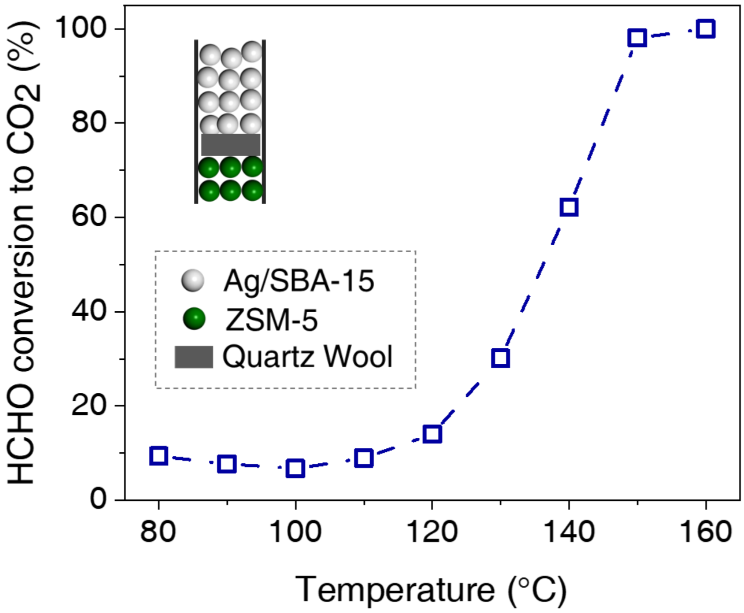

Supplementary Figure 5. Reaction performance of Ag/SBA-15–ZSM-5 composite. ZSM-5_46 was packed below 10Ag/SBA-15 layer at a distance of 3 mm separated by inert quartz wool. Reaction conditions: the mass ratio of ZSM-5_46 to 10Ag/SBA-15 is 1/4, 100 ppm HCHO, 20% O_2-Ar, 36000 mL g^{-1} h^{-1}.

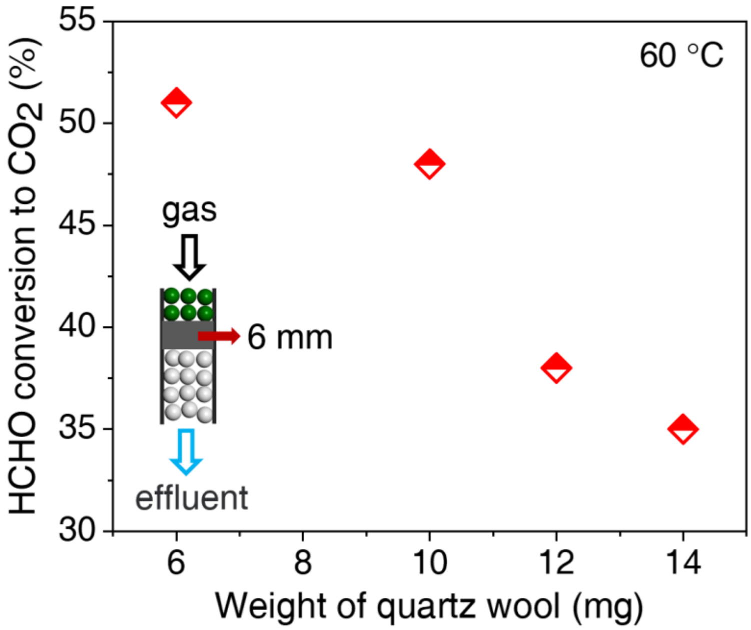

Supplementary Figure 6. Mass transfer-dependent catalytic activity. The distance between the two layers is kept at 6 mm, and the density of quartz wool is changed by adjusting its weight. Reaction conditions: 60 °C, mass ratio of ZSM-5_46 to 10Ag/SBA-15 is 1/4, 100 ppm HCHO, 20% O_2-Ar, 36000 mL g^{-1} h^{-1}.

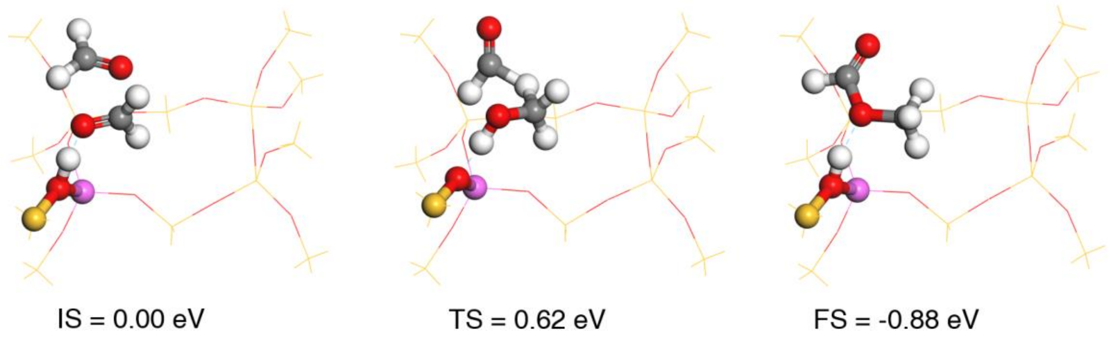

Supplementary Figure 7. Geometries and free energies. The initial state (IS), transition state (TS), and final state (FS) for HCHO conversion to $HCOOCH_3$ (methyl formate, MF) over acidic ZSM-5 zeolite. All energies are referred to initial state. The white, gray, red, purple, and yellow atoms refer to H, C, O, Al and Si, respectively.

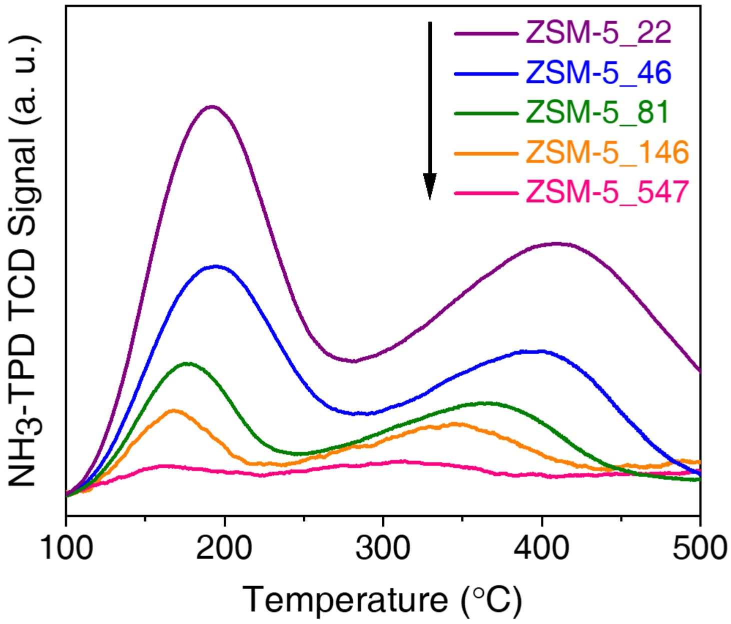

Supplementary Figure 8. NH_3-TPD profiles of zeolites. ZSM-5 with different densities of acid sites.

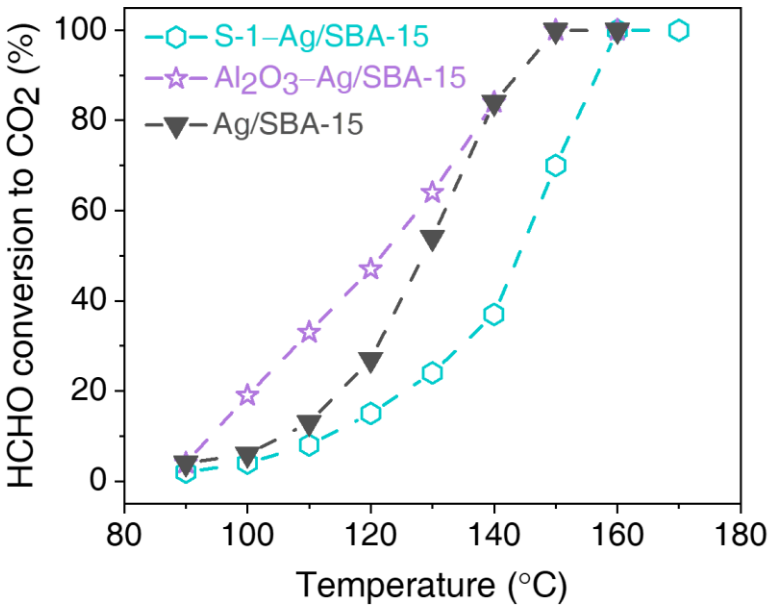

Supplementary Figure 9. Reaction performance of HCHO conversion over S-1–Ag/SBA-15 and Al_2O_3–Ag/SBA-15. Activity of Ag/SBA-15 was presented as a comparison. Reaction conditions: the mass ratio of S-1 or Al_2O_3 to 10Ag/SBA-15 is 1/4, 100 ppm HCHO, 20% O_2-Ar, 36000 mL g^{-1} h^{-1}.

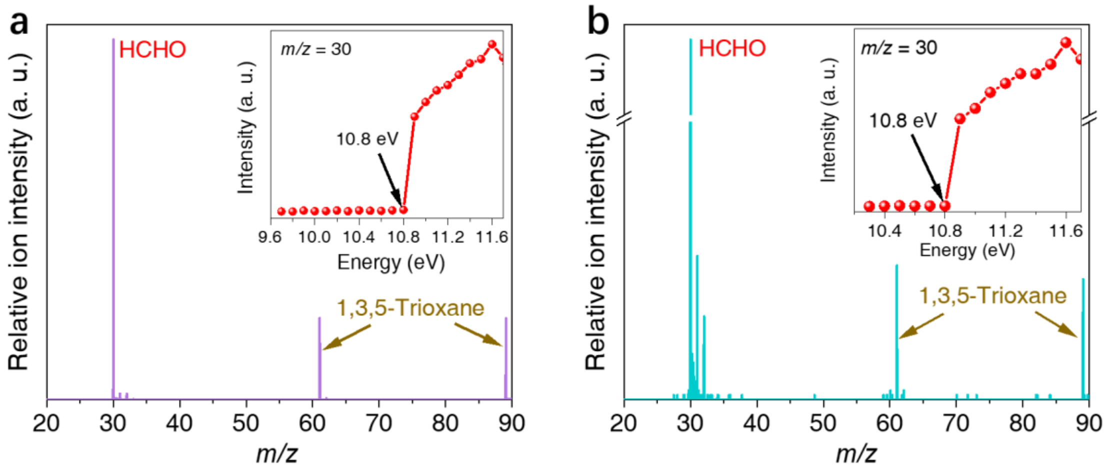

Supplementary Figure 10. *In-situ* study of HCHO conversion by SVUV-PIMS at the photon energy of 11.3 eV. a S-1 sample. **b** Al_2O_3 sample. Inset is the PIE spectrum of *m/z* = 30 species of HCHO. Reaction conditions: 2 torr, 65 ^{o}C and GHSV = 21000 mL g^{-1} h^{-1}.

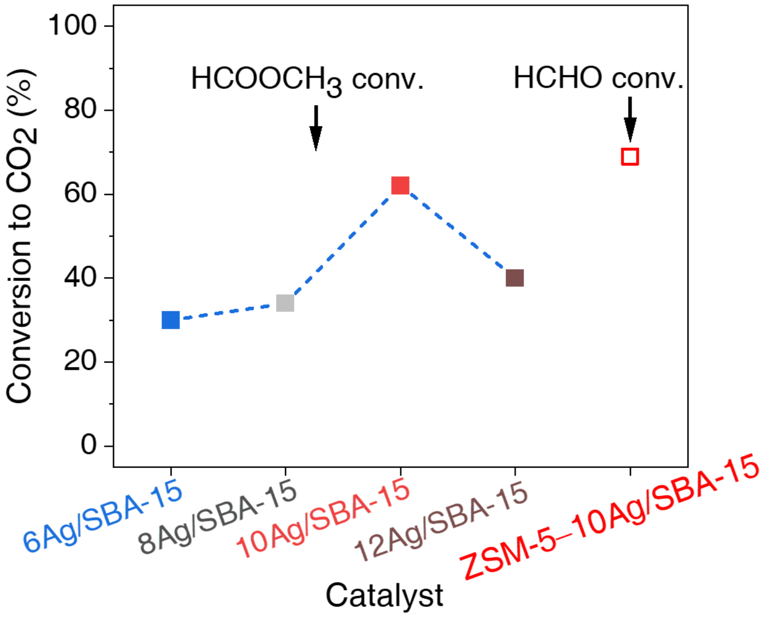

Supplementary Figure 11. Reaction performance of model experiments. $HCOOCH_3$ and HCHO conversion over different catalysts at a constant temperature of 60 °C.

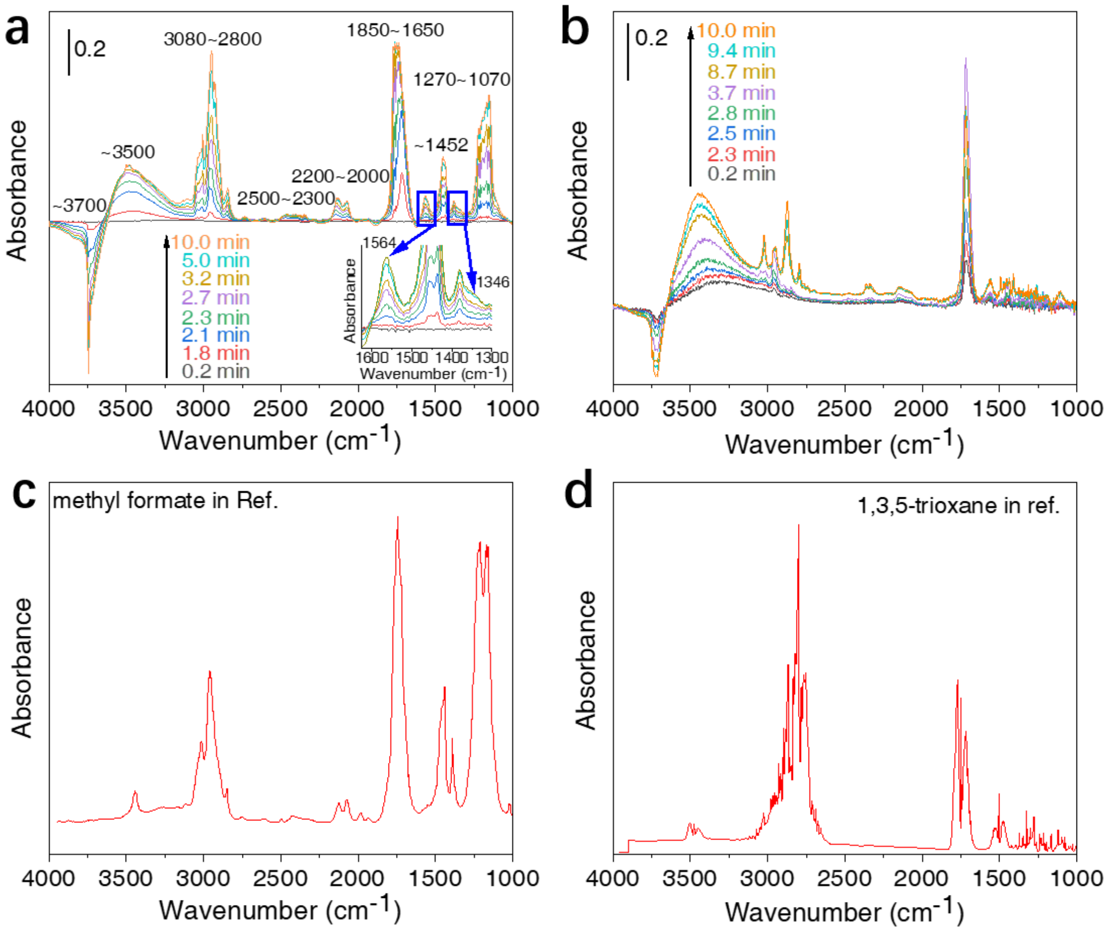

Supplementary Figure 12. *In-situ* DRIFT spectra of reactants adsorption processes. a $HCOOCH_3$ and **b** HCHO adsorption on Ag/SBA-15 catalyst, respectively. Standard IR spectra of **c** $HCOOCH_3$ (methyl formate, HR Nicolet Sampler Library) and **d** 1,3,5-trioxane (Aldrich FT-IR Collection Edition II, Aldrich Catalog no. T8110-8, CAS number: 110-88-3).

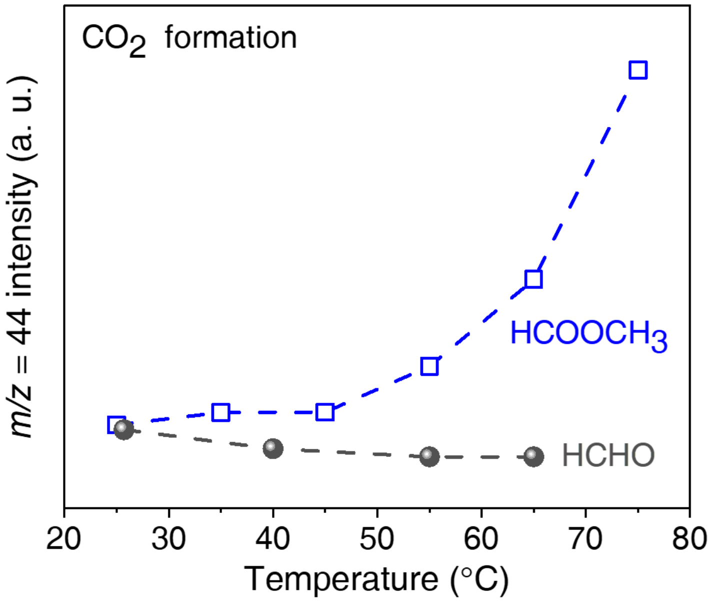

Supplementary Figure 13. Model experiments of HCHO and $HCOOCH_3$ conversion to CO_2 (*m*/*z* = 44) over 10Ag/SBA-15. Intensities were monitored by an online quadrupole mass spectrometer.

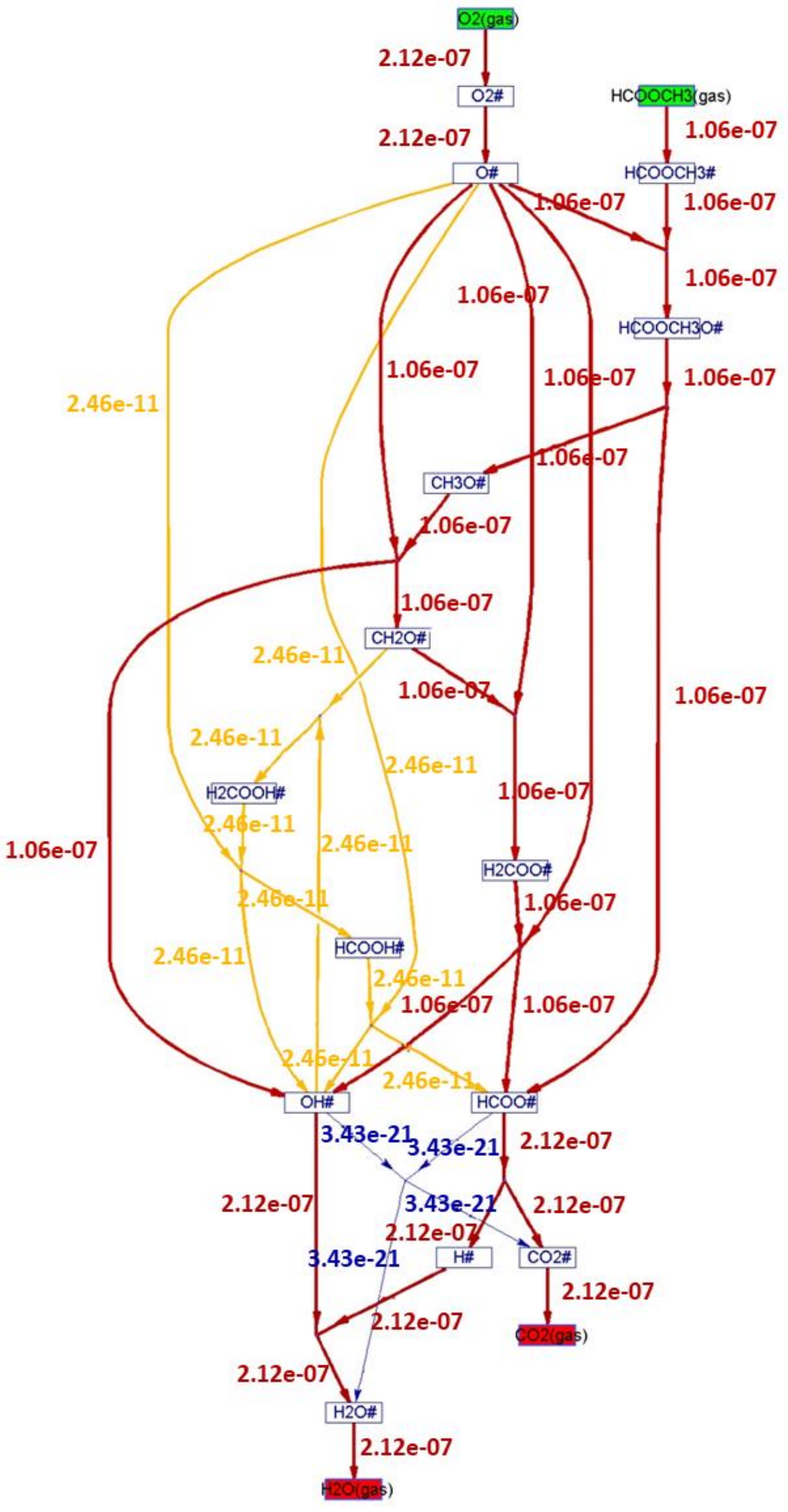

Supplementary Figure 14. A flow chat for $HCOOCH_3$ oxidation over Ag(111). The number indicates the reaction rate (TOF) in exponential scale. The symbol # indicates an adsorption site on the surface.

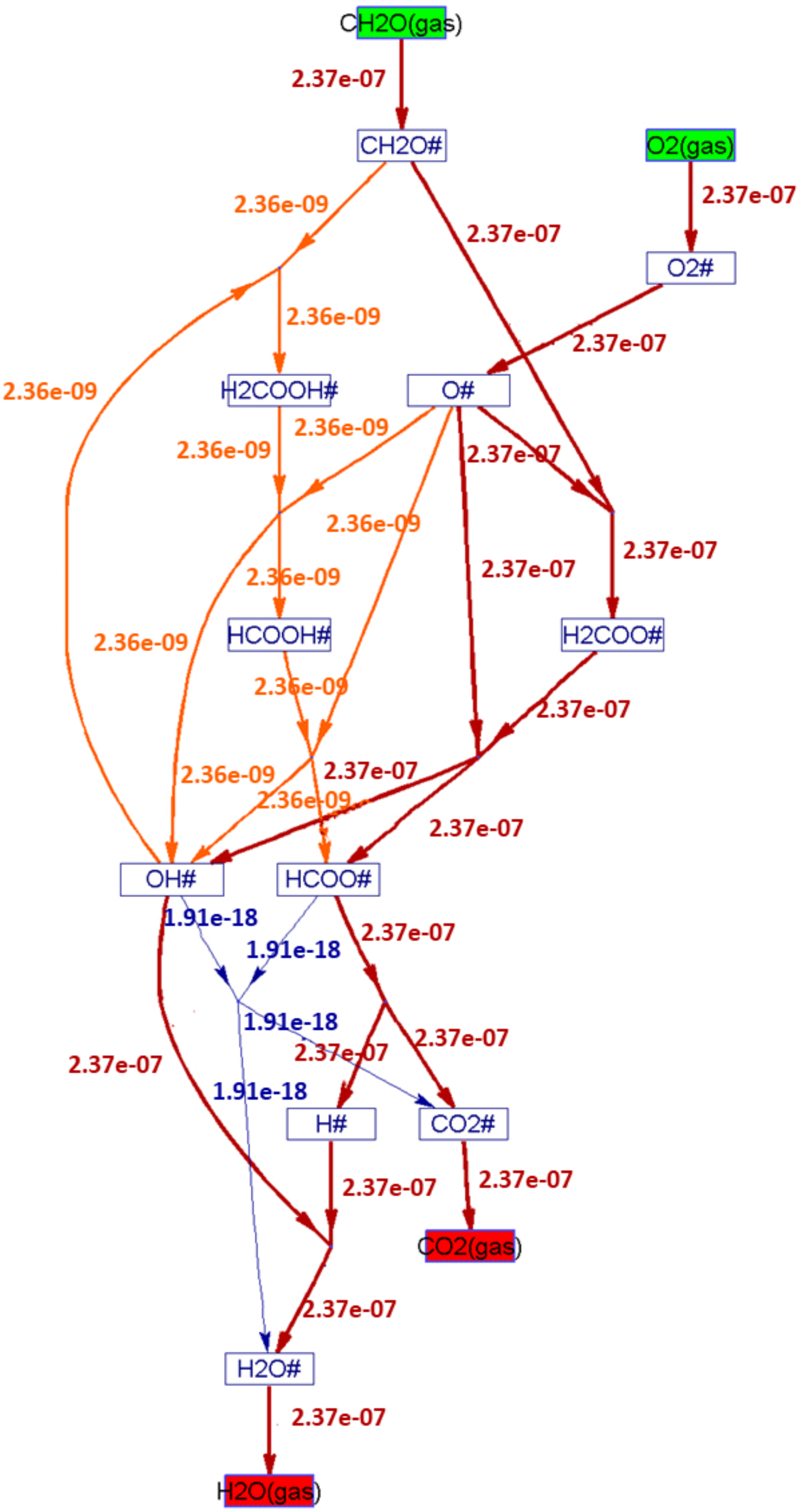

Supplementary Figure 15. A flow chat for HCHO oxidation over Ag(111). The number indicates the reaction rate (TOF) in exponential scale. The symbol # indicates an adsorption site on the surface.

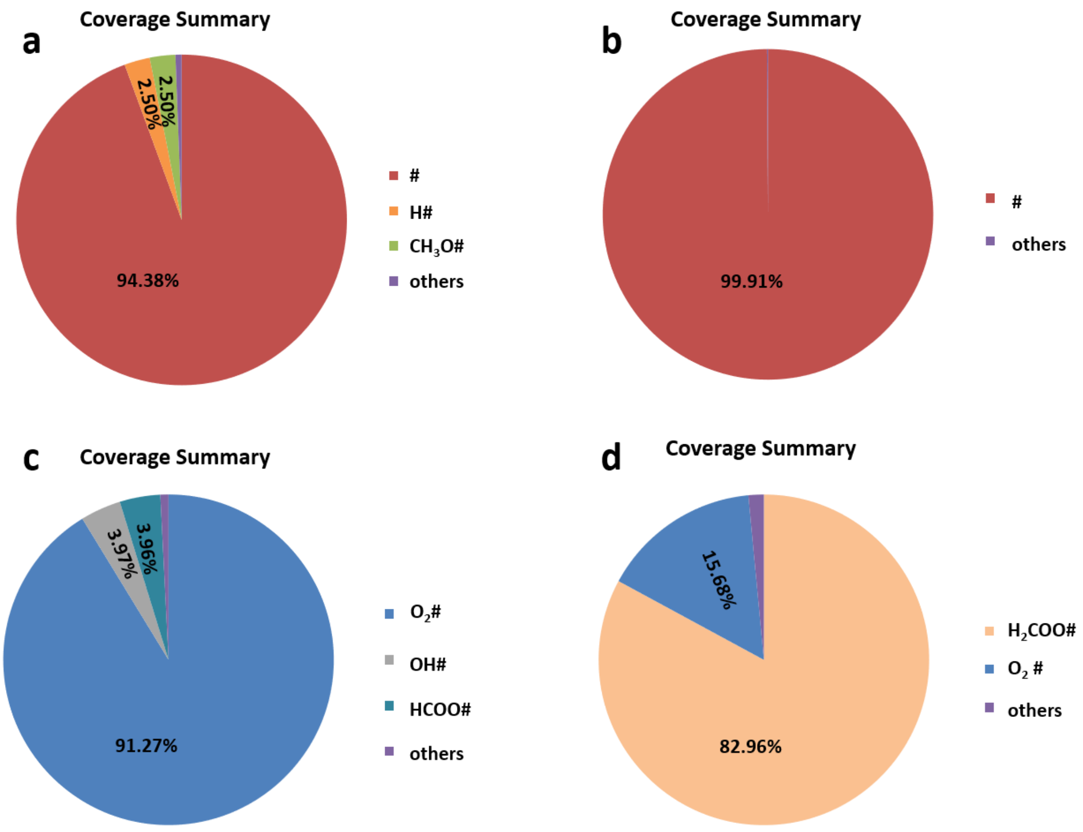

Supplementary Figure 16. Coverage summary at the steady state. **a** $HCOOCH_3$ oxidation on Ag(111). **b** HCHO oxidation on Ag(111). **c** $HCOOCH_3$ oxidation on Ag(100). **d** HCHO oxidation on Ag(100). The symbol # refers to the free sites on the surface.

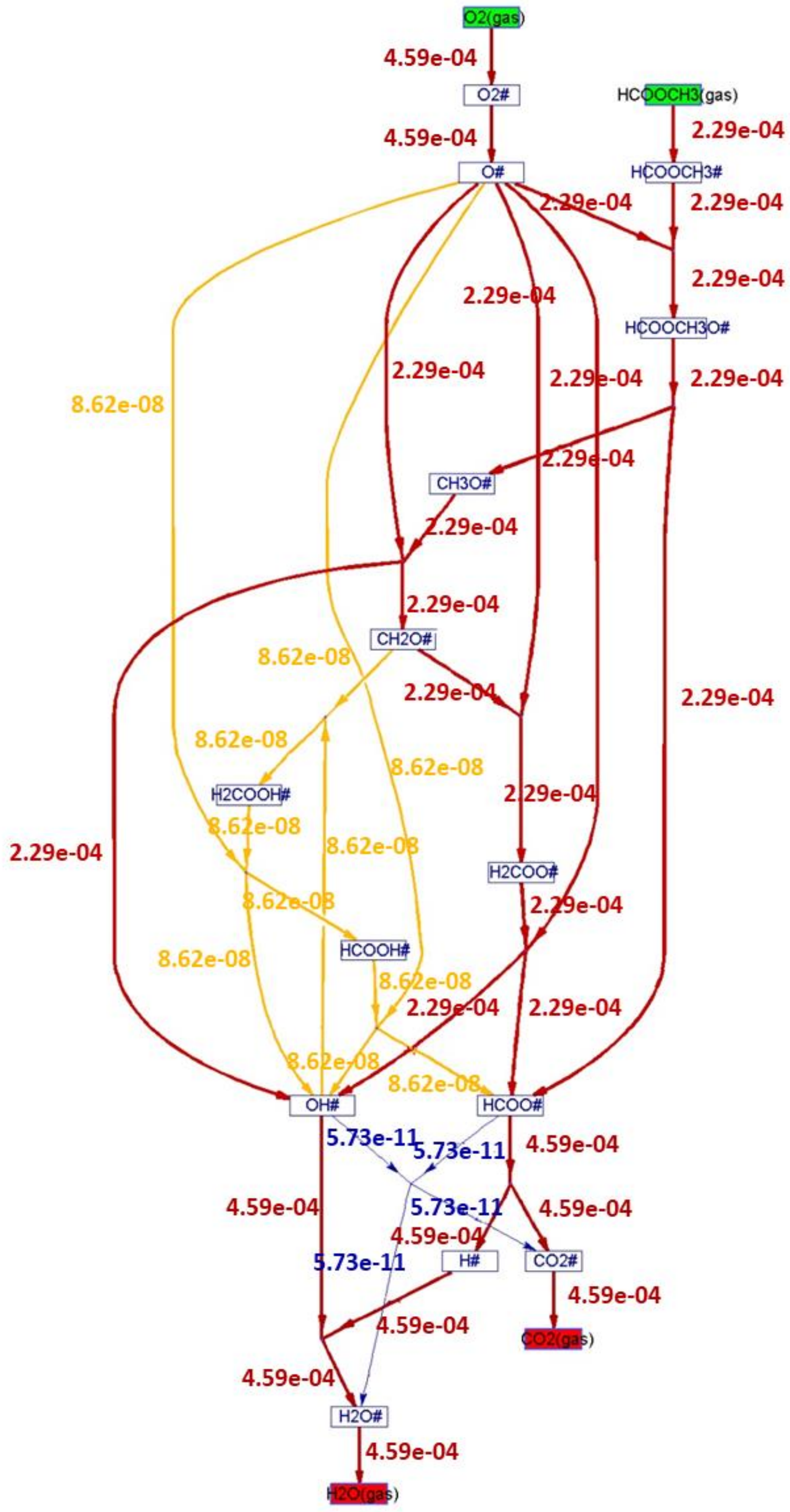

Supplementary Figure 17. A flow chat for $HCOOCH_3$ oxidation over Ag(100). The number indicates the reaction rate (TOF) in exponential scale. The symbol # indicates an adsorption site on the surface.

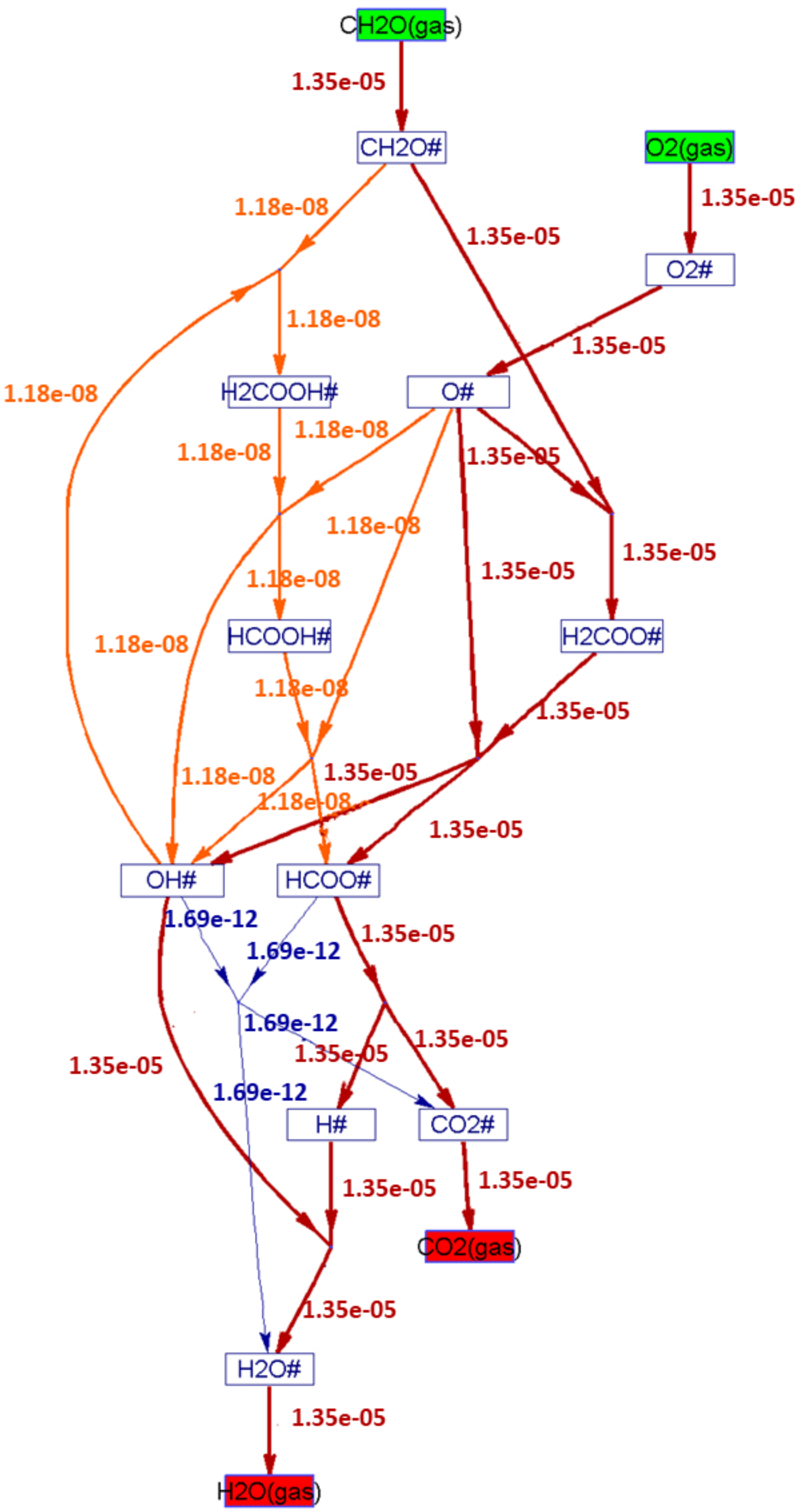

Supplementary Figure 18. A flow chat for HCHO oxidation over Ag(100). The number indicates the reaction rate (TOF) in exponential scale. The symbol # indicates an adsorption site on the surface.

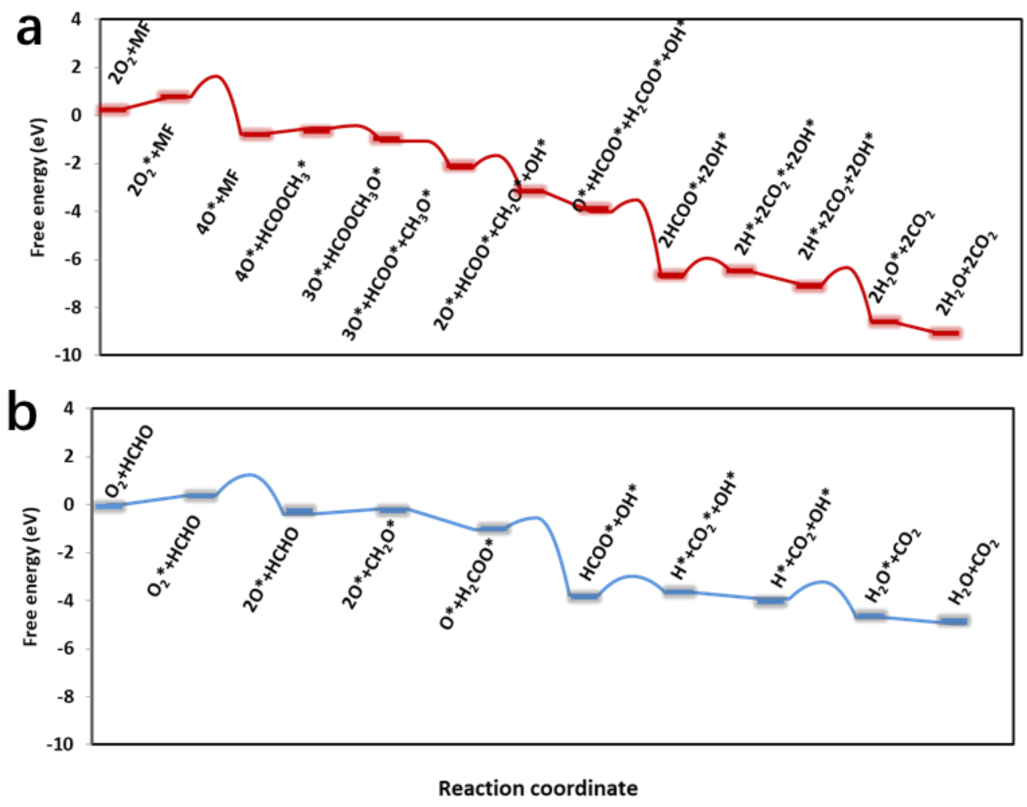

Supplementary Figure 19. Free energy diagrams on Ag(111) surface. a $HCOOCH_3$ (red line) and **b** HCHO (blue line) oxidation processes.

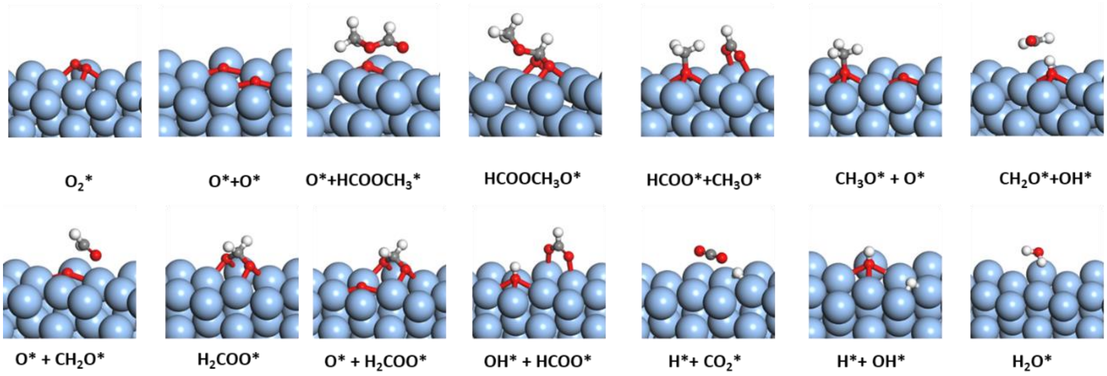

Supplementary Figure 20. The optimized structures of the initial and final states for $HCOOCH_3$ and HCHO oxidation over Ag(100). The Ag, C, O, and H are represented in silver, grey, red, and white.

Supplementary Table 1. Infrared band assignments of the surface species.

Surface species	Vibrations	Wavenumbers (cm^{-1})	References
Formate	$\upsilon_{as, COO} + \delta_{CH}$	2950	[1-8]
	υ_{CH}	2831	
	$\upsilon_{as, COO}$	1564	
	$\upsilon_{s, COO}$	1346	
Methyl formate	υ_{CH3}	2962	[1, 4, 9, 10]
	υ_{CH3O}	2846	
	$\upsilon_{C=O}$	1715	
	$\delta_{as, CH3}$	1452	
	$\delta_{s, CH3}$	1437	
	γ_{CH3}	1219-1153	
Formaldehyde	$\upsilon_{s, CH2}$	2795	[4-7, 11, 12]
	$\upsilon_{C=O}$	1720	
	δ_{CH2}	1495	
Dioxymethylene	υ_{CH2}	2872	
	δ_{CH2}	1474	
	ω_{CH2}	1410	
	υ_{C-O}	1107	
CO_2		2359, 2341	[8]
H_2O		1615	[6]
Surface hydroxyl	υ_{OH}	3700	[7, 9]

Supplementary Table 2. Calculated barriers (G_a) and reaction energies (ΔG) of considered elementary steps. HCHO and $HCOOCH_3$ oxidation on Ag(111) and Ag(100) surfaces, respectively.

	Reaction	Ag(111)		Ag(100)	
		G_a	ΔG	G_a	ΔG
R1	O_2 + # ↔ O_2#	0.39	0.39	0.00	-0.29
R2	O_2# + # ↔ 2O#	0.85	-0.78	0.82	-1.27
R3	$HCOOCH_3$ + # ↔ $HCOOCH_3$#	0.25	0.25	0.28	0.28
R4	$HCOOCH_3$# + O# ↔ $HCOOCH_3O$# + #	0.10	-0.54	0.12	-0.60
R5	$HCOOCH_3O$# + # ↔ HCOO# + CH_3O#	0.00	-1.13	0.21	-1.17
R6	CH_3O# + O# ↔ OH# + CH_2O#	0.53	-0.95	0.50	-0.47
R7	CH_2O# + O# ↔ H_2COO# + #	0.00	-0.86	0.00	-0.86
R8	H_2COO# + O# ↔ OH# + HCOO#	0.49	-2.73	0.77	-2.28
R9	HCOO# + # ↔ H# + CO_2#	0.79	0.11	0.73	0.64
R10	H# + OH# ↔ H_2O# + #	0.70	-0.78	0.68	-0.55
R11	H_2O# ↔ H_2O + #	0.00	-0.25	0.00	-0.11
R12	CO_2# ↔ CO_2 + #	0.00	-0.26	0.00	-0.40
R13	CH_2O# + OH# ↔ H_2COOH# + #	0.00	-0.27	0.15	-0.18
R14	H_2COOH# + O# ↔ HCOOH# + OH#	0.33	-1.64	0.56	-1.19
R15	HCOOH# + O# ↔ HCOO# + OH#	0.00	-1.67	0.00	-1.77
R16	HCOO# + OH# ↔ H_2O# + CO_2#	1.23	-0.66	1.34	0.09
R17	CH_2O + # ↔ CH_2O#	0.21	0.21	0.19	0.19

R3-R6 are not related to HCHO oxidation and are not included in the microkinetic modelling of HCHO oxidation. R17 is not related to $HCOOCH_3$ oxidation and is not included in the microkinetic modelling of $HCOOCH_3$ oxidation. The symbol # indicates an adsorption site on the surface.

Supplementary Table 3. Degree of rate control (X_{RC}) of certain elementary steps on $HCOOCH_3$ oxidation rate over Ag(100). The symbol # indicates an adsorption site on the surface.

Reaction	X_{RC}
O_2# + # ↔ 2O#	0.83
HCOO# + # ↔ H# + CO_2#	0.16
H_2COO# + O# ↔ OH# + HCOO#	0.01

The X_{RC} values of the other elementary reaction steps related to $HCOOCH_3$ oxidation in Supplementary Table 2 are close to 0.00 and therefore not listed.

Supplementary Table 4. Degree of rate control (X_{RC}) of certain elementary steps on HCHO oxidation rate over Ag(100). The symbol # indicates an adsorption site on the surface.

Reaction	X_{RC}
O_2# + # ↔ 2O#	0.37
CH_2O# + O# ↔ H_2COO# + #	-0.46
H_2COO# + O# ↔ OH# + HCOO#	1.06
HCOO# + # ↔ H# + CO_2#	0.03

The X_{RC} values of the other elementary reaction steps related to HCHO oxidation in Supplementary Table 2 are close to 0.00 and therefore not listed.

Supplementary References

1. Millar, G. J., Rochester, C. H.&Waugh, K. C. Infrared study of methyl formate and formaldehyde adsorption on reduced and oxidised silica-supported copper catalysts. *J. Chem. Soc., Faraday Trans.* **87**, 2785-2793 (1991).

2. Millar, G. J., Metson, J. B., Bowmaker, G. A.&Cooney, R. P. An in situ Fourier transform Infrared study of formic acid adsorption on a polycrystalline silver catalyst. *J. Catal.* **147**, 404-416 (1994).

3. Millar, G. J., Metson, J. B., Bowmaker, G. A.&Cooney, R. P. Influence of oxidation and reduction conditions upon the morphology of silica-supported polycrystalline silver catalysts. *J. Chem. Soc., Faraday Trans.* **91**, 133-139 (1995).

4. Popova, G. Y., Andrushkevich, T. V., Chesalov, Y. A.&Stoyanov, E. S. In situ FTIR study of the adsorption of formaldehyde, formic acid, and methyl formiate at the surface of TiO_2 (anatase). *Kinet. Catal.* **41**, 805-811 (2000).

5. Xu, B., Zhu, T., Tang, X.&Shang, J. Heterogeneous reaction of formaldehyde on the surface of TiO_2 particles. *SCI China Chem.* **53**, 2644-2651 (2010).

6. Shi, C., et al. Catalytic formaldehyde removal by "storage-oxidation" cycling process over supported silver catalysts. *Chem. Eng. J.* **200**, 729-737 (2012).

7. Zhu, X., Yu, J., Jiang, C.&Cheng, B. Catalytic decomposition and mechanism of formaldehyde over Pt-Al_2O_3 molecular sieves at room temperature. *Phys. Chem. Chem. Phys.* **19**, 6957-6963 (2017).

8. Ou, C. C., Chen, C. H., Chan, T. S., Chen, C. S.&Cheng, S. Influence of pretreatment on the catalytic performance of Ag/CeO_2 for formaldehyde removal at low temperature. *J. Catal.* **380**, 43-54 (2019).

9. Chuang, C. C., Wu, W. C., Huang, M. C., Huang, I. C.&Lin, J. L. FTIR study of adsorption and reactions of methyl formate on powdered TiO_2. *J. Catal.* **185**, 423-434 (1999).

10. Gazsi, A., Schubert, G., Pusztai, P.&Solymosi, F. Photocatalytic decomposition of formic acid and methyl formate on TiO_2 doped with N and promoted with Au. Production of H_2. *Int. J. Hydrogen Energ.* **38**, 7756-7766 (2013).

11. Millar, G. J., Rochester, C. H.&Waugh, K. C. An FTIR study of the adsorption of formic acid and formaldehyde on potassium-promoted Cu/SiO_2 catalysts. *J. Catal.* **155**, 52-58 (1995).

12. Rasko, J., Kecskes, T.&Kiss, J. Adsorption and reaction of formaldehyde on TiO_2-supported Rh catalysts studied by FTIR and mass spectrometry. *J. Catal.* **226**, 183-191 (2004).
